# Flexible Optoelectronic Hybrid Microfiber Long‐period Grating Multimodal Sensor

**DOI:** 10.1002/advs.202501352

**Published:** 2025-03-08

**Authors:** Zhenru Li, Li‐Peng Sun, Yanzhen Tan, Zhiwei Wang, Xiao Yang, Tiansheng Huang, Jie Li, Yi Zhang, Bai‐Ou Guan

**Affiliations:** ^1^ Guangdong Provincial Key Laboratory of Optical Fiber Sensing and Communications Institute of Photonics Technology College of Physics & Optoelectronic Engineering Jinan University Guangzhou 510632 China; ^2^ School of Electronic Engineering and Intelligentization Dongguan University of Technology Dongguan 523808 China; ^3^ Key Laboratory of Biomaterials of Guangdong Higher Education Institutes Department of Biomedical Engineering Jinan University Guangzhou 510632 China

**Keywords:** hybrid multimodal sensor, laser‐induced graphene, microfiber long‐period grating, optoelectronic device

## Abstract

Flexible wearable biosensors have emerged as a promising tool for tracking dynamic glycemic profiles of human body in diabetes management. However, it remains a challenge to balance the shrunken device space and multiple redundant sensing arrays for further advancement in miniaturization of multimodal sensors. Herein, this work proposes an entirely new optoelectronic hybrid multimodal optical fiber sensor which is composed of laser patterning of polydimethylsiloxane (PDMS) to form laser‐induced graphene (LIG) as the interdigital electrodes, and a long period grating (LPG) prepared from an optical microfiber encapsulated into the PDMS modulated by periodical structure of LIG electrodes. This operation can simultaneously integrate two heterogeneous sensing mechanisms, optical and electrical, into a single sensor in a compact manner. Combining the LIG electrode with conductive hydrogel, a flexible glucose biosensor based on electrical mechanism is constructed by loading glucose oxidase into the hydrogel. Meanwhile, the microfiber LPG can also be served as a spectroscopically available sensor for biomechanical monitoring. Optical and electrical sensors can work simultaneously but independently of each other, particularly in the scene of wound healing for rat model and movement for human exercise. This platform represents a pivotal step toward multifunctional sensors that enable measurements of biomechanical information and glucose.

## Graphical abstract

An entirely new optoelectronic hybrid multimodal optical fiber sensor which can simultaneously monitor biomechanics and glucose is demonstrated. The developed method simultaneously integrates two heterogeneous sensing mechanisms, optical and electrical, into a single sensor in a compact manner. Optical and electrical sensors can work simultaneously but independently of each other, particularly in the scene of wound healing for rat model and movement for human model.

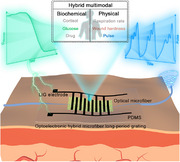

## 1 Introduction

Hyperglycemia forms a threatening risk factor for mortality and disability worldwide.^[^ ^1^ ^]^ With the rising trend of diabetic population worldwide, exploring and interpreting the complex nature of human wellness to prognosticate glucose metabolism disorder and diagnose the diabetes have become an urgent task in the concern of human health.^[^ ^2^, ^3^, ^4^ ^]^ The abnormal glucose concentration induced by insulin imbalance haunts the patients since diabetes will cause a series of complications such as foot infection which may beget amputation in severe cases, and jeopardize patients’ eyes, kidneys, blood vessels, heart and other organs. Conventional glucose measurements mainly rely on the invasive and intermittent acupuncture blood testing, which exists a latency between testing and evaluation and forms a trepidation to the patients. Such measurements have difficulties in providing rapid monitoring of dynamic glycemic levels in case of sudden changes in the physiological condition.

The emergence of wearable biosensors exhibited astounding potential in the realms of continuous non‐invasive diabetes management, where alternative body fluids such as interstitial fluid, sweat, saliva and tears were exploited for glucose monitoring.^[^ ^5^, ^6^, ^7^, ^8^, ^9^ ^]^ Specifically, by on‐site sample analysis of human sweat, pulse, movement behavior, and other biochemical or physical changes, wearable biosensors can endow efficient and advanced manners for unobtrusive monitoring of chronic diseases and are beneficial for pathophysiological abnormalities assessment during daily activities of wearers.^[^ ^10^, ^11^, ^12^, ^13^ ^]^ Over the past decades, wearable biosensor platforms have been developed and their functionality has shifted to measure multiple biochemical and physical health parameters since one single parameter analysis might lead to misdiagnosis. An integrated sensing array with an enzyme‐based current sensor and a potential sensor was proposed to simultaneously detect sweat metabolites, electrolytes and skin temperature.^[^ ^14^ ^]^ This pioneering work offered a promising paradigm for the subsequent research on multiplexed platforms for numerous diabetes biomarkers detection.^[^ ^15^, ^16^, ^17^, ^18^ ^]^ However, most current studies for biomarkers detection are either based on redundant sensing arrays, which are not conducive to sensor integration and miniaturization, or based on single component multimodal sensor, which often requires complex algorithms for decoupling due to signal correlation under a single mechanism to ensure reliable data output for each detection quantity.^[^ ^19^ ^]^ On the other hand, simultaneously capturing vital biomechanical or physical parameters such as heart rate, blood pressure, and monitoring wound healing status in daily activity, can provide a comprehensive assessment of a human health status and aid healthcare professionals in critical decision‐making. These have triggered the development of hybrid multimodal biosensors with different self‐decoupled mechanisms, which can optimize the multifunctional capability of simultaneous perception on both biochemical and physical parameters and effectively reduce the signal interference.^[^ ^20^, ^21^, ^22^ ^]^ For example, a hybrid multimodal wearable array was proposed by integrating an ultrasonic transducer and an electrochemical sensor into a unitary patch thanks to the advancement in fabrication and miniaturization of the bio‐electronic components, which can coinstantaneously measure glucose, caffeine, alcohol, blood pressure and heart rate, with reduced crosstalk between two sensing modalities.^[^ ^23^ ^]^ This methodology is versatile and these results also provide a new insight for multivariate bio‐signal measurements. Nevertheless, accommodating multiple sensing modalities into a single wearable platform also faces challenges in sensors integration and compatibility between different manufacturing technologies. Especially, in the aspect of identifying multifarious output data by considering complex fusion and cross‐modality precision, which would form higher complexity for the miniaturization of electronics. Balancing the relationship between shrunken device space and multiple redundant sensing arrays requires efficient device designs and strategies, and advanced fabrication techniques for next‐generation multimodal biosensors. Therefore, novel methods that can construct highly integrated heterogeneous biosensing devices for accurate biochemical and physical monitoring, crucially need to be developed.

**Figure 1:**
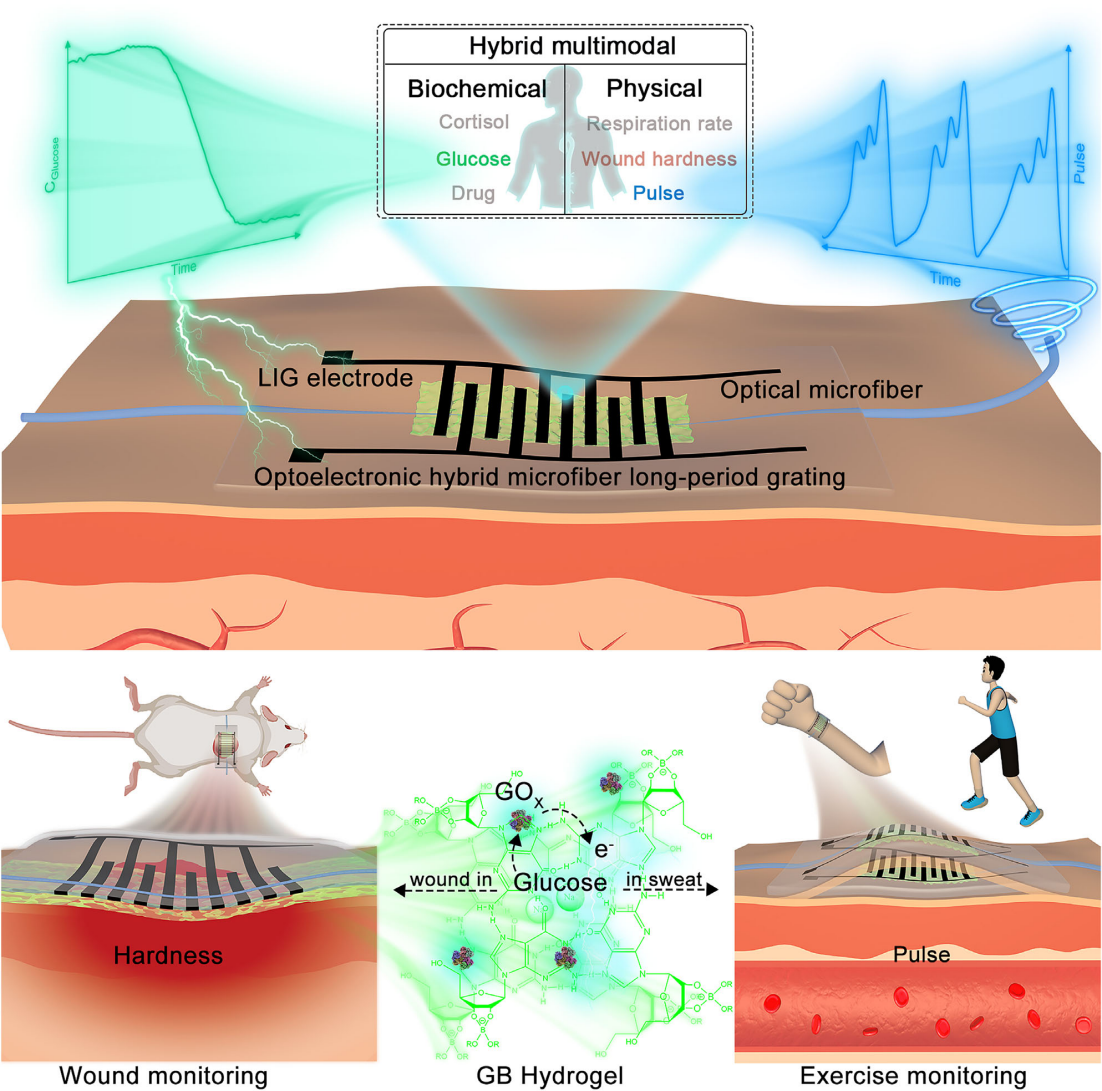
The schematic illustration of LIG‐mLPG flexible optoelectronic hybrid multimodal sensor. Inset: The diagram of functionalized LIG‐mLPG for monitoring sweat glucose and physical quantities in animal and human models.

Constructing multidimensional structures on flexible substrates has provided an advanced technique for wearable sensors or devices.^[^ ^24^, ^25^, ^26^, ^27^, ^28^, ^29^, ^30^ ^]^ In this study, we demonstrated an entirely new and biocompatible optoelectronic hybrid multimodal sensor that is composed of laser patterning of polydimethylsiloxane (PDMS) to form laser‐induced graphene (LIG) as the interdigital electrodes, and a long period fiber grating prepared from an optical microfiber encapsulated into PDMS modulated by periodic refractive index of the interdigital structure, as shown in **Figure** **1**. To address the issues of redundant sensing design and signal interference in current multimodal sensors, we integrated two heterogeneous sensing mechanisms, optical and electrical, into a single sensor device by the advanced LIG technology, thus ensuring that two signals operate in a separate domain and work independently of each other. Combined with the conductive hydrogel, LIG can be used as a glucose biosensor based on electrical signal. Meanwhile, the microfiber long period grating (mLPG) triggers mode coupling with resonant wavelength, which can be concurrently served as an spectral sensor for biomechanical monitoring. More importantly, we thoroughly study and verify the independence of two sensing mechanisms upon different stimuli, and further demonstrate the monitoring of hardness and glucose of diabetes wound healing in rat as well as artery pulse and sweat glucose of human exercise. This laser‐induced graphene‐microfiber long period grating (LIG‐mLPG) flexible optoelectronic hybrid multimodal sensor offers a new design concept for compact and multifunctional wearable sensors that enable reliable measurements for various hybrid stimuli monitoring and provides better understanding on glucose levels and human biomechanical parameters for optimized management of diabetes.

## 2 Results and Discussion

### 2.1 Design and Fabrication of LIG‐mLPG

**Figure 2:**
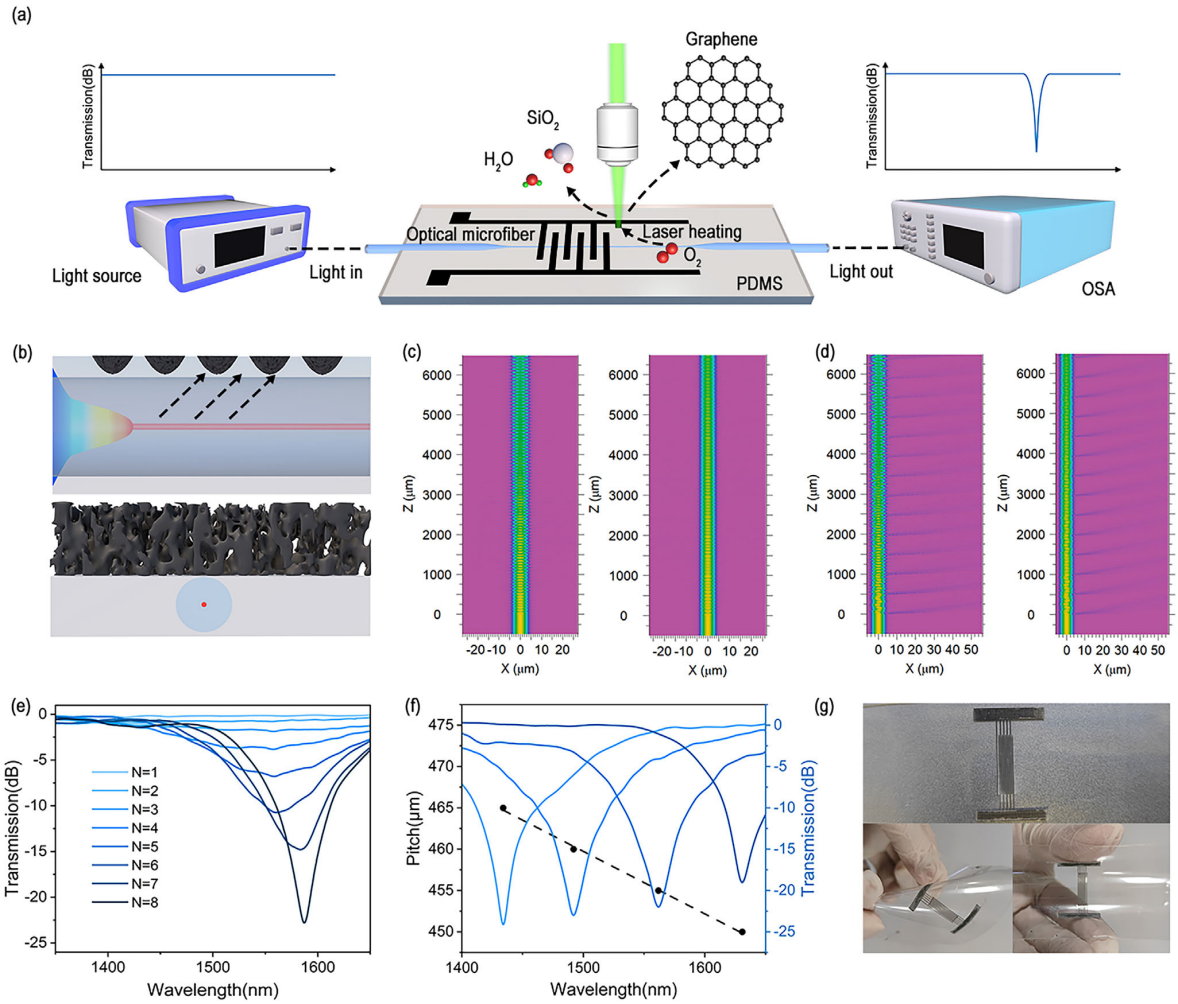
a) Conceptual diagram of mLPG based on laser‐induced graphene modulation; b) Top: axial structural diagram of LIG‐mLPG; Bottom: cross‐sectional diagram of LIG‐mLPG; c) Top view and d) side view of the simulated amplitude distribution of light propagating through the grating region when the light is launched at resonant (phase‐matching, left panel) and non‐resonant (non‐matching, right panel) wavelengths, respectively; e) Evolution of transmission spectra changes with modulation period number during LIG‐mLPG modulation process; f) Relationship between the pitch of grating and resonant wavelength; g) Physical image of LIG‐mLPG.

The specific configuration of the LIG‐mLPG and the conceptual diagram of the device fabrication system are illustrated in **Figure** **2**. To overcome the problems of permanent damage such as deformation or even melting of the microfibers induced by high energy consumption from lasers or arc pulses, a mLPG was designed by a novel LIG modulation technique as an efficiently wearable sensor for hybrid multimodal parameters measurement. Before laser operation, a microfiber was firstly drawn to ∼8 µm from standard single mode fiber (SMF) by using the flame‐brushing technique to amplify the energy distribution of the evanescent field.^[^ ^31^ ^]^ Such tapered microfiber was then encapsulated into a PDMS substrate by specific fabrication in the Experimental Section and Figure S1, Supporting Information. Subsequently, the PDMS was placed under the laser focal plane. The PDMS absorbed the incident laser energy and converted it into thermal heat and was then transformed into porous graphene under photothermal action. The interdigital structure of the patterned porous graphene in PDMS forms periodic modulation in the evanescent field of microfibers, ultimately producing a long‐period grating, as shown in Figure 2a. The corresponding axial structural and cross‐sectional diagrams of the fabricated LIG‐mLPG are shown in Figure 2b, giving that the generated patterning of porous graphene is also served as the mask‐like modulation for the mLPG, creating new optoelectronic integrated devices. Meanwhile, LIG pattern periodically modulates the leaked evanescent field of the microfiber, rather than directly contact the microfiber, which offers a novel and efficient method for mLPG fabrication. When the incident light transmits through the LIG‐mLPG, the HE_12_ mode is excited and coupled with the fundamental HE_11_ mode along the propagation direction. Figure 2c,d show the top view and side view, respectively, of the simulated amplitude distribution of light propagating through the grating region when the light is launched at resonant (1550 nm, left panel) and non‐resonant (1250 nm, right panel) wavelengths. It can be observed that the optical field undergoes a strong perturbation when the light passes through the grating region under resonant condition. Such field perturbation results from the periodic refractive index modulation in the fiber grating, which is known as the phase‐matching condition.^[^ ^32^ ^]^ Figure 2e shows the measured evolution spectrum of the prepared LIG‐mLPG with the period number N ranging from 1 to 8, where the depths of resonant dip increased from 0 to 22.5 dB, indicating that the coupling strength increases with the modulation number. Meanwhile, Figure 2f demonstrates the recorded phase‐matching relationship (grating pitch‐resonant wavelength) between the fundamental HE_11_ and HE_12_ modes of the LIG‐mLPG, manifesting that the resonant wavelength of the mLPG can be flexibly designed through changing the grating pitch of the mLPG. Figure 2g shows the physical image of the fabricated LIG‐mLPG, exhibiting an excellent flexibility. Such characteristics of mLPG and PDMS make the device promising for flexible sensing applications, such as measuring the pressure and deformation.

### 2.2 Characterization of the LIG‐mLPG

**Figure 3:**
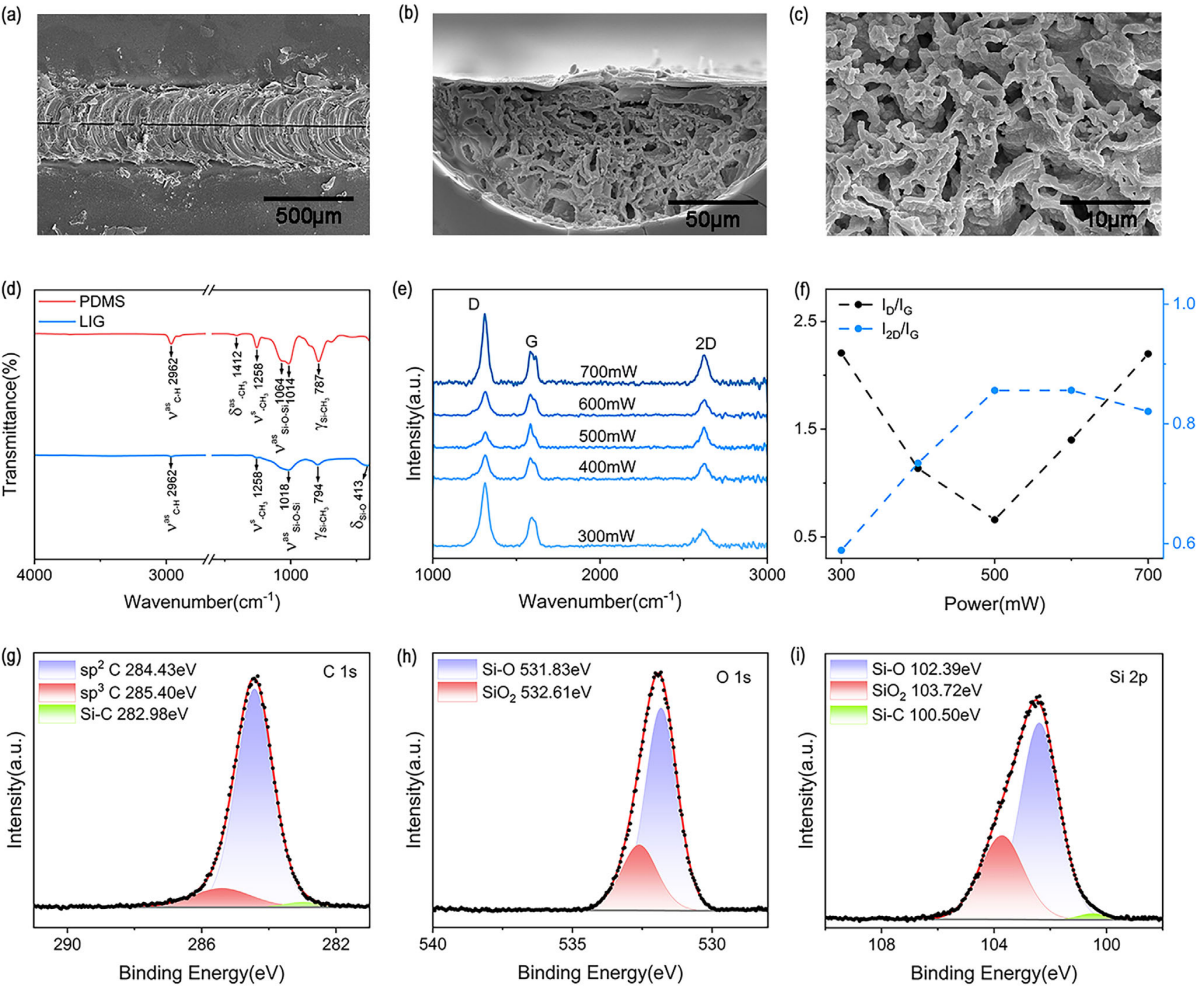
SEM images of a) the top view and b) cross section of the LIG; c) Enlarged SEM image of cross‐section of the LIG; d) FTIR of PDMS (red) and LIG (blue); e) Raman spectrum of LIG under different modulation powers; f) I_D_/I_G_ (black) and I_2D_/I_G_ (blue) change with laser modulation power; XPS of LIG at g) C 1s; h) O 1s; i) Si 2p.

Specifically, by irradiating the PDMS, chemical bonds break due to pyrolysis and photolysis, leading to the formation of volatile hydrocarbons. With sufficient energy, these hydrocarbons can rearrange and recombine to form aromatic carbon structures, resulting in graphitic carbon.^[^ ^33^ ^]^ Therefore, the modulation effect of the LIG‐mLPG strongly depends on the laser power and scanning speed during the patterning process.^[^ ^34^ ^]^ The width, height, composition, and morphology of the produced graphene were analyzed under different laser powers and scanning speeds via scanning electron microscope (SEM), X‐ray photoelectron spectroscopy (XPS), Raman spectroscopy, and Fourier Transform Infrared Spectrometer (FTIR Spectrometer) studies. As the laser power increases and the scanning speed decreases, the accumulated heat will increase, expanding the size of graphene in terms of the width and depth (Figures S3 and S4, Supporting Information). It is evident from the SEMs of LIG in **Figure** 3a–c that a multilayer graphite carbon structure forms after the escape of gaseous products during the laser patterning process.^[^ ^35^ ^]^ Figure 3d displays the FTIR spectrum of PDMS before (red line) and after (blue line) laser induction. After laser induction, the asymmetric stretching vibration peak representing methyl at 2962 cm^−1^, the asymmetric deformation peak representing methyl at 1412 cm^−1^, the symmetric deformation peak representing methyl at 1258 cm^−1^, and the strong absorption peak representing Si‐CH_3_ vibration at 787 cm^−1^ all decrease after the formation of LIG. The asymmetric stretching vibration peak and asymmetric deformation peak of Si‐O‐Si located at 1064 and 1014 cm^−1^ merge into one peak at 1018 cm^−1^, as well as the appearance of the Si‐O peak at 413 cm^−1^, which are related to the silica nanoparticles.^[^ ^36^ ^]^ These results all indicate that the Si‐C, Si‐O, and C‐H bonds in PDMS are broken after laser irradiation. The silicon and hydrogen atoms subsequently combine with the oxygen atoms in the air or in PDMS to constitute silicon dioxide and gaseous water, while the remaining carbon atoms become the porous graphene structures through the carbonization and graphitization processes.^[^ ^37^ ^]^

In order to produce high‐quality graphene, we further characterized the graphene films with laser power ranging from 300 to 700 mW via Raman spectroscopy, as shown in Figure 3e. The Raman spectra were obtained in the range of 1000–3000 cm^−1^. As seen in the spectra, LIG was formed after laser‐induced modification since the D, G, and 2D Raman peaks of graphene were clearly observed, which are located at the wavelength of 1350, 1580, and 2700 cm^−1^, respectively. When the laser power increased from 300 to 700 mW, there was no significant change in peak position, however, the relative intensities of three Raman peaks of graphene varied, indicating that the quality of produced graphene strongly depends on the laser power. Specifically, in Figure 3f, the ratio of I_D_ peak to I_G_ peak (I_D_/I_G_) first decreases and then increases whereas the ratio of I_2D_ peak to I_G_ peak (I_2D_/I_G_), representing the number of graphene layers, shows an opposite trend with the rising laser power.^[^ ^38^ ^]^ These results can be attributed to the higher localized temperature of PDMS films with increasing laser power, which is conducive to produce high‐quality porous graphene when the laser power increases from 300 to 500 mW. However, the I_D_/I_G_ becomes strong remarkably and the I_2D_/I_G_ decreases gradually when the laser power further increases to 700 mW, implying the presence of more defects in the LIG due to the oxidation of graphene at excessive temperature.^[^ ^37^ ^]^ We also obtained similar results in Raman spectroscopy at different scanning speeds (Figure S5, Supporting Information). Within an appropriate range, the higher the scanning speed, the better the quality of the obtained graphene.

We characterized the graphene with XPS when the laser power and the scanning speed are 500 mW and 2mm s^−1^, respectively. The high‐resolution XPS spectra of C 1s region for graphite are shown in Figure 3g, the deconvoluted C1s peaks show three binding energies of 284.43, 285.40 and 282.98 eV, representing C‐C (sp^2^), C‐C (sp^3^), and Si‐C bonds, respectively.^[^ ^39^, ^40^ ^]^ In Figure 3h, the deconvoluted O 1s peaks show two different peaks located at 531.83 and 532.61 eV, which are ascribed to Si‐O and SiO_2_, respectively.^[^ ^41^, ^42^ ^]^ In Figure 3i, the states observed at 100.50, 102.39, and 103.72 eV are related to Si‐C, Si‐O, and SiO_2_, respectively.^[^ ^40^, ^43^ ^]^ From XPS spectra and FTIR spectrum, it was inferred that the chemical bonds of PDMS were broken and recombined to form graphene.

### 2.3 Sensor Performance

#### 2.3.1 Pressure Sensing Performance of the Multimodal Sensor

**Figure 4:**
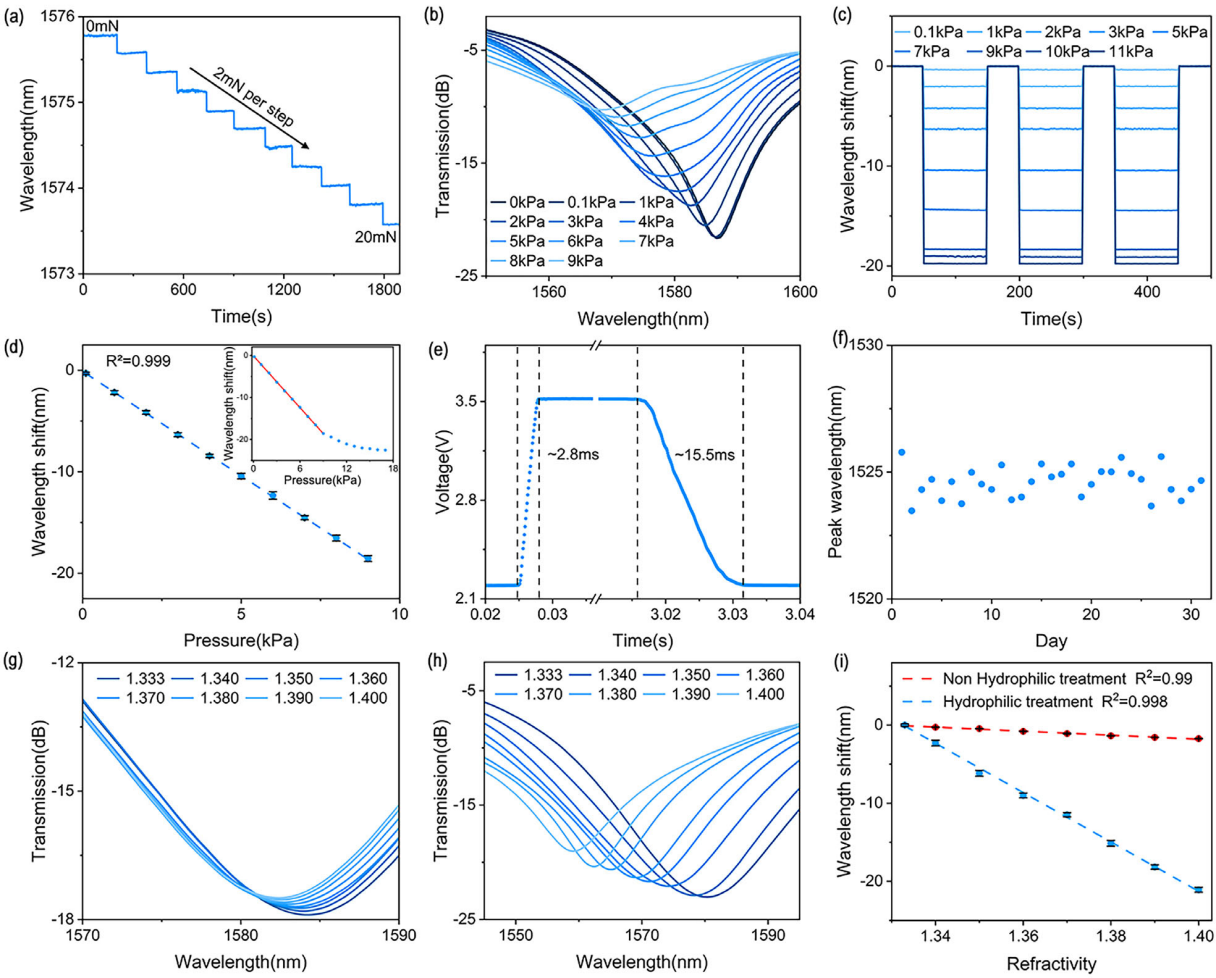
a) Measured dip wavelength shift of mLPG with a stress step of 2 mN; b) Recorded transmission spectra of a single dip at different pressure levels; c) Repeatability of LIG‐mLPG wavelength shift under different pressure levels; d) Measured dip wavelength shift with the applied pressure; e) The response time and recovery time of LIG‐mLPG under 1 kPa pressure; f) The stability of LIG‐mLPG within a month; Recorded transmission spectra of a single dip under different refractive index g) before and h) after hydrophilic treatment; i) Measured dip wavelength shift with refractive index before and after hydrophilic treatment.

The biomechanical sensing ability of the hybrid multimodal sensor is implemented by the optical signal of LIG‐mLPG, where the pressure level is encoded into wavelength spectral change. To investigate the pressure sensitivity of the proposed LIG‐mLPG, different values of normal stress from a digital push‐pull force tester were applied to the grating. LIG‐mLPG deformed under normal stress, changing the grating matching conditions and causing a shift of the resonant wavelength. The dip of resonant wavelength shift of the LIG‐mLPG with the normal stress changing from 0 to 20 mN was recorded and shown in **Figure** **4**a, and it shows a clear response to a pressure of 2 mN per step and a high signal‐to‐noise ratio. The transmission spectra of a single dip under different pressure are shown in Figure 4b, and the resonant wavelength gradually decreases with increasing pressure. The wavelength shift of LIG‐mLPG under different pressure was also measured and shown in Figure 4c, which manifests a good repeatability. The wavelength shift caused by pressure was calibrated within the measurable range from 0.1–17 kPa (inset) in Figure 4d. The pressure sensitivity is defined as S = (W_t_‐W_0_)/ΔP, where ΔP represents the pressure change, W_t_ is the wavelength corresponding to the pressure change, W_0_ is the initial wavelength. The measured sensitivity was estimated as 2.06 nm kPa^−1^ by linearly fitting with the external pressure from 0.1 to 9 kPa, and the detection limit is 0.008 kPa. As shown in Figure 4e, the response time and recovery time of LIG‐mLPG under 1 kPa pressure were ∼2.8 and 15.5 ms, respectively. Therefore, the prepared LIG‐mLPG not only has high sensitivity and linear range to the pressure, but also shows fast response. Finally, LIG‐mLPG also showed good stability in a stationary state with a wavelength drift of <15 pm within 1 h and a standard deviation of ∼2.6 pm (Figure S6, Supporting Information). It remained stable for a month with a spectral fluctuation standard deviation of ∼0.608 nm, as shown in Figure 4f.

We further validated the refractive index (RI) response of the LIG‐mLPG by immersing the LIG‐mLPG into the sodium chloride solution with different concentrations, and the corresponding RI changed from 1.333 to 1.4. The grating spectral response was shown in Figure 4g, exhibiting a low sensitivity of 25.8 nm/RIU. This is due to the hydrophobicity of LIG‐mLPG, with a hydrophobic angle of 125.72° (Figure S7, Supporting Information). Then we performed hydrophilic operation on LIG‐mLPG where its hydrophobic angle becomes 66.9° (Figure S7, Supporting Information). After hydrophilic operation, its refractive index spectral response with RI sensitivity of 314.5 nm/RIU is shown in Figure 4h. Figure 4i shows the comparison of the refractive index response, showing good linearity and giving ∼12.19 times improvement after hydrophilic operation. Therefore, we can achieve physical and biochemical parameters measurement through flexible interface modification. Here, we choose a low refractive index sensitivity design to minimize the influence of external biochemical parameters. Therefore, the optical response of the device can be served as a sensing element which is biomechanical sensitive but insensitive to biomarkers due to the high hydrophobicity of LIG‐mLPG with RI sensitivity of only 25.8 nm/RIU in liquid environments.

#### 2.3.2 Electrical Response of Graphene Electrode in the Multimodal Sensor

**Figure 5:**
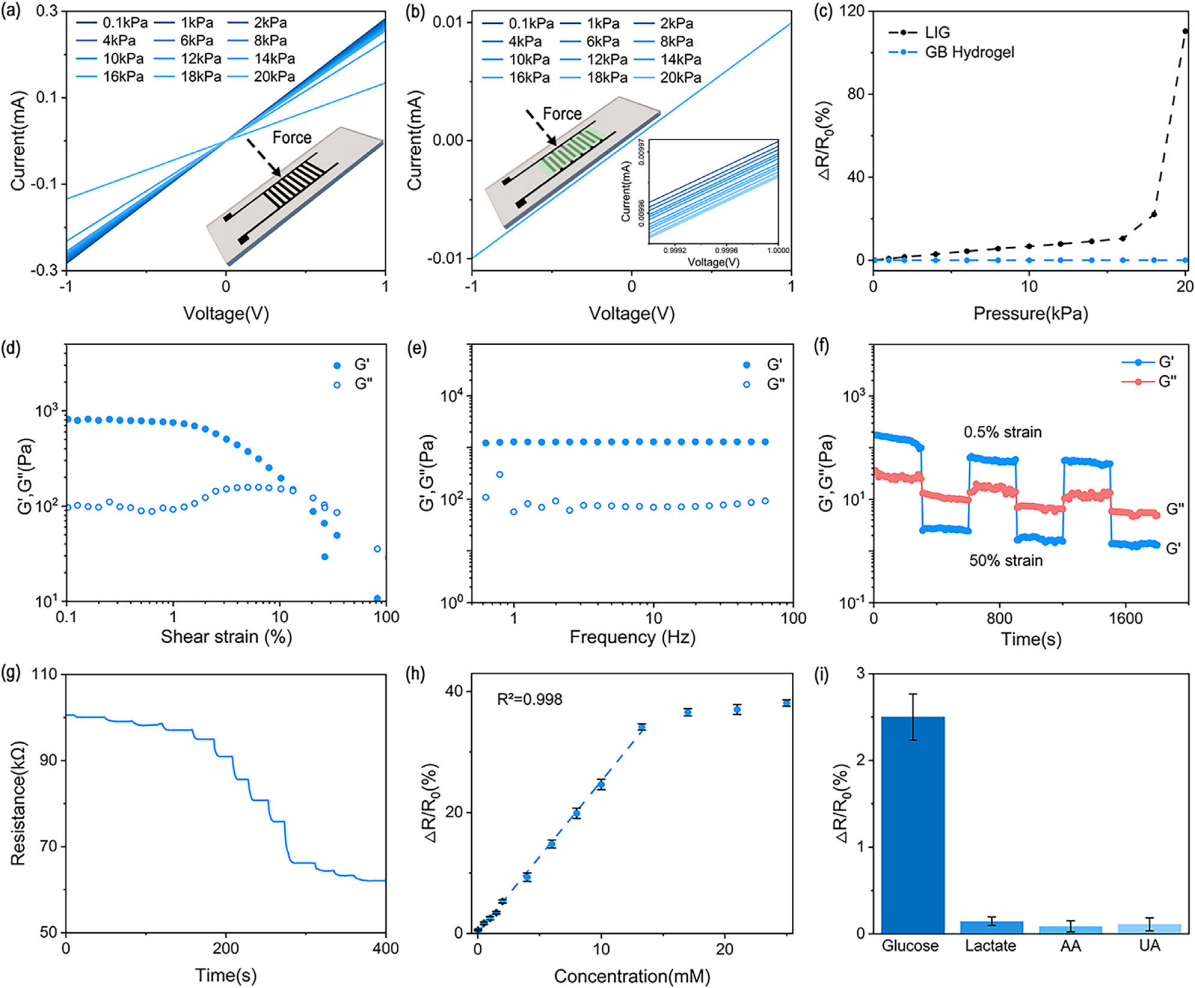
a) I‐V characteristics of LIG electrode (positive and negative electrodes connected) within 20 kPa; b) I‐V characteristics of GB hydrogel/LIG (interdigital electrode structure) within 20 kPa; c) Comparison of resistance changes between the positive and negative connection pattern of LIG and the GB hydrogel/LIG under different pressures; The storage modulus G′ and loss modulus G″ of GB hydrogel change under different d) shear strains and e) frequencies; f) Rheological recovery test performed with alternately switched shear strain between large strain (50%) and small strain (0.5%); g) The resistance changes of the GB hydrogel doped with GOx under different glucose concentrations (0–25 mM); h) Resistance changes with different glucose concentration; i) Specificity for glucose.

The biochemical sensing part of the hybrid multimodal sensor is implemented by electrical signals of LIG interdigital electrode, where the glucose concentration is encoded into the resistance. We first tested the conductivity of graphene electrodes via CV and EIS tests (Figures S8 and S9, Supporting Information), and the results showed that graphene electrodes have good charge transfer ability and can be used as electrical sensing components. Additionally, we tested the stability of the electrode resistance, which also showed good stability in a stationary state. The resistance change was less than 0.02% within 2 h, with a standard deviation of ∼0.003%, and remained stable for 1 month with a standard deviation of ∼0.965 Ω (Figure S10, Supporting Information). To achieve a strain insensitive chemical detection platform, we introduced GB hydrogel with good conductivity and biocompatibility (structure as shown Figure S11, Supporting Information).^[^ ^44^ ^]^ As illustrated in **Figure** **5**a, the I‐V characteristics changed with different values of pressure when using LIG directly as the sensing electrode. This may be attributed to the cracks in the LIG caused by pressure deformation, leading to changes in resistance.^[^ ^45^ ^]^ In contrast, no significant change was observed in the I‐V curve under different pressure after introducing the GB hydrogel in Figure 5b. This is because the conductive hydrogel compensates the broken LIG cracks through external circuit to maintain its conductivity under high pressure deformation, which enhances its continuous conductive tensile ability.^[^ ^46^ ^]^ The strain sensitivity of a resistor is defined as S = (ΔR/R_0_)/ΔP, where ΔP is the pressure change, ΔR = R_0_‐R_t_, R_t_ is the resistance under the corresponding pressure change, R_0_ is the initial resistance. In Figure 5c, the strain sensitivity of LIG was measured to be ∼0.657%/kPa from 0 to 16 kPa, however, when it exceeds 16 kPa, the strain crack increases abruptly and the resistance increases sharply. After combining with the GB hydrogel, the strain sensitivity is only ∼0.003%/kPa with the pressure changing from 0.1–20 kPa, and the resistance change under small pressure deformation can be ignored. Due to the characteristics of hydrogel, we can achieve strain insensitivity of the LIG‐mLPG. In the rheological characteristics of hydrogel, the storage modulus G′ remained higher than its loss modulus G″ in the shear strain range from 0.1% to 10%, as shown in Figure 5d, which indicated that GB hydrogel has good viscoelasticity and the ability to resist the large shear strain. The shear strain value of 1% in the linear viscoelastic region was selected for further frequency scanning and the result are shown in Figure 5e. Both G′ and G″ exhibit weak frequency dependence within 1–60 Hz and maintain good mechanical properties (G′ > G″). The GB hydrogel experiences the transition from gel to sol state (G″ > G′) when the strain becomes 50% from 0.5%, whereas the G′ is higher than G″ when the strain is quickly switched back to 0.5%, as shown in Figure 5f, indicating that the GB hydrogel has the ability of rapid self‐healing and can repair these structures through the non‐covalent and dynamic chemical bonds when mechanical strain occurs. Moreover, in the rheological test with different fluid environments and glucose in different concentrations, GB hydrogel maintains good mechanical response (G′ > G″) (Figure S12, Supporting Information). As shown in Figure 5g, after loading the GOx into GB hydrogel, the resistance gradually decreases when the glucose concentration increases from 0 to 25 mM, which is due to the decomposition of glucose and the generation of electrons under the action of GOx.^[^ ^47^ ^]^ Furthermore, the byproducts of hydrogen peroxide and gluconic acid both destroy the structure of GB hydrogel and increase the conductivity, thus amplifying the resistance change during glucose decomposition to a certain extent. Figure 5h shows the measured resistance changes with different concentrations of glucose, and indicates a linear range of 0.02 mM ∼13.28 mM with the detection limit of ∼0.0246 mM. Due to the existence of GOx, the hydrogel has good specificity for glucose detection, as shown in Figure 5i. These results show that the GB hydrogel could be used as a strain insensitive and flexible material in sensor for glucose detection in the low frequency range of practical detection. In addition, to verify the insensitivity of grating optical signal to glucose variation, we detected the response of LIG‐mLPG to different concentrations of glucose. The result showed that the grating spectral signal remained almost unchanged when glucose was less than 50 mM (Figure S13, Supporting Information). And in 4000 times stability test under 4 kPa, both the optical and electrical sensors maintained good repeatability (Figures S14 and S15, Supporting Information). Therefore, LIG‐mLPG combined with GB hydrogel can be regarded as a biosensor for simultaneous monitoring of glucose and biomechanical parameters in the biological fluid environments.

### 2.4 Detection of Diabetes Wound Healing Using the LIG‐mLPG Sensor

**Figure 6:**
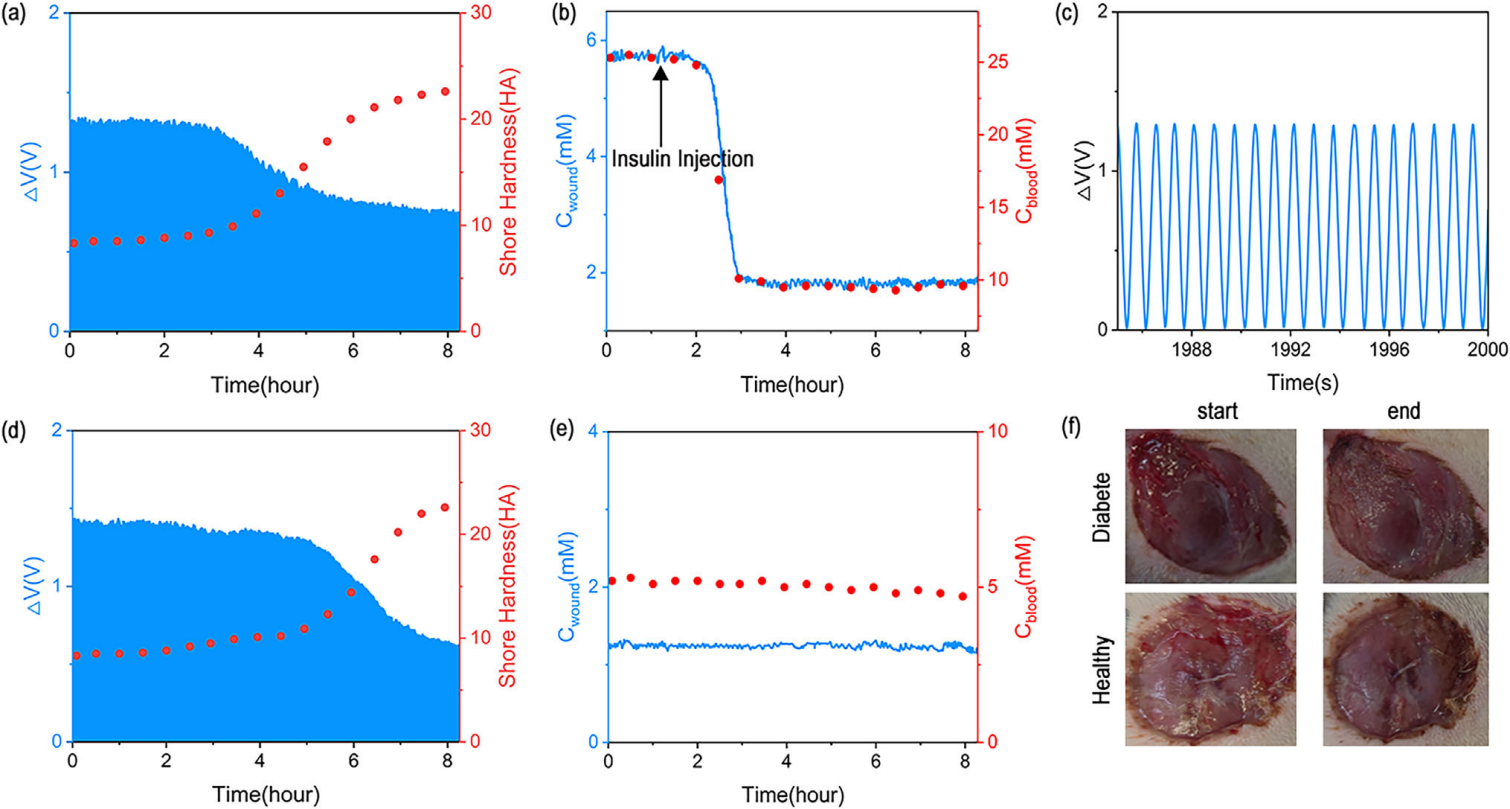
a) The monitoring of wound of the diabetic rat in fiber grating signal (blue) and the change in wound hardness measured by commercial Shore hardness tester (red); b) The monitoring of wound of the diabetic rat in hydrogel electrical signal (blue) and the change in blood glucose measured by commercial blood glucose meters; c) Respiratory signal of the diabetic rat (magnified view of the grating signal in a); d) The monitoring of wound of the healthy rat in fiber grating signal (blue) and the change in wound hardness measured by commercial Shore hardness tester (red); e) The monitoring of wound of the healthy rat in hydrogel electrical signal (blue) and the change in blood glucose measured by commercial blood glucose meters; f) Changes in the wound at the beginning and end of the monitoring.

Wound healing in diabetes is a complex and dynamic process, involving real‐time monitoring of wound environment, early identification of infection risk and accurate assessment of healing progress.^[^ ^48^ ^]^ In this section, we explore the feasibility of the multimodal sensor for monitoring the wound hardness and glucose level in diabetic wound healing process. We first investigated the spectral response of the LIG‐mLPG sensor to the hardness by conformally attaching the LIG‐mLPG to the objects with different hardness. The results are shown in Figure S16, Supporting Information, which indicates the greater the hardness, the smaller the spectral response. After that, the LIG‐mLPG was adhered to the wound near the abdomen of rats with diabetes. The abdomen undulation will deform the device due to the periodic respiratory activity, the hardness during the wound healing process can be monitored by the sensor. **Figure** **6**a shows that the wound gradually heals and its hardness increases with time.^[^ ^49^ ^]^ The signal variation of LIG‐mLPG induced by respiratory deformation gradually decreases (blue curves), which is in agreement with the change in wound hardness measured by a commercial shore hardness tester (red dots). As revealed in Figure 6b, the resistance signal (electrical signal) of the GB hydrogel was captured to measure the glucose concentration in the wound exudate. During the monitoring process, we injected the insulin into the rats, then the level of glucose concentration began to decline. The trend of this changes measured throughout the entire process (blue curves) is consistent with the blood glucose changes measured with a commercially available blood glucose meter (red dots). Figure 6c shows the respiratory signal of the diabetic rat within the time slot of 15 s, which indicated that the respiratory signals can also be captured when monitoring the hardness of the wound. Similarly, the LIG‐mLPG was attached to the wound of healthy rats, and the trend of wound healing process was similar to that of diabetes rats, as shown in Figure 6d. It was found that the hardness gradually increased as the wound slowly healed. The variation tendency was also consistent with the change of wound hardness measured with a commercial shore hardness tester. Since the healthy rats were not injected with insulin when monitoring, the electrical signal measured by the sensor had no significant drift or degradation, given that the glucose concentration in the wound exudate was proven to be basically stable. The change trend was also consistent with that measured by the commercial glucose meter, as shown in Figure 6e. Figure 6f shows the photos of the wound changes before and after measurement for the diabetes rats and healthy rats.

### 2.5 Real‐Time Multiparameter Monitoring of Human Health

**Figure 7:**
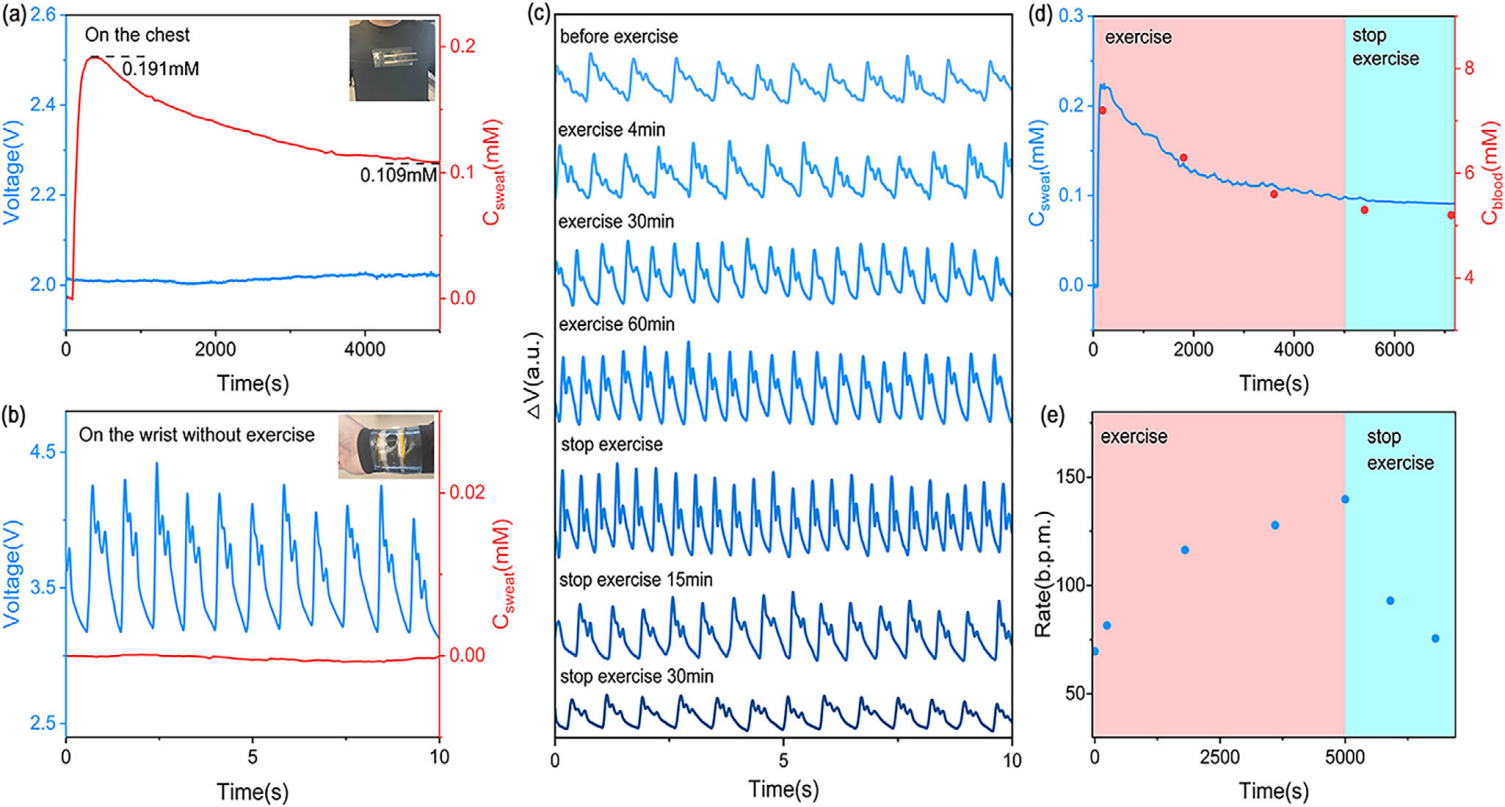
a) Monitoring on the chest during exercise (no deformation, only sweat); b) Monitoring on the wrist at rest (no sweat, deformation caused by pulse); c) Changes in the pulse wave on the wrist during exercise; d) Changes in glucose concentration in sweat (blue) and blood glucose measurement with a commercial blood glucose meter (red) during exercise; e) Changes in pulse rate over time.

Biomechanical information such as heart rate, artery pulse, and blood pressure are important vital signs of the human body. Simultaneous monitoring of such biomechanical and biochemical information can provide a comprehensive assessment of a human health status, help identify some potential concurrent diseases, facilitate early intervention, and better manage diabetes. Benefitting from the high flexibility and multimodal perception abilities, we investigate the feasibility of the multimodal sensor for simultaneous monitoring the pulse and sweat glucose signals by attaching LIG‐mLPG onto the surface of tight sportswear during daily exercise. To verify the independence of optical and electrical signals from the LIG‐mLPG, a healthy volunteer wore the LIG‐mLPG onto his chest in motion and to the wrist in a stationary state, respectively. As shown in **Figure** **7**a, the volunteer began to move and the sweat flowed out as the movement continued, leading to glucose detection (no deformation, only sweat). Due to high calorie consumption with continuous exercise, the concentration of glucose slowly decreases from 0.191 mM to 0.109 mM.^[^ ^50^ ^]^ However, there is no superficial artery at the chest, so no pulse signal can be captured by the sensor. In contrast, when LIG‐mLPG is attached near the superficial artery of the wrist, the contour of the periodic pulse wave can be clearly detected in a stationary state without movement, but there is no sweat flowing out in the stationary state, so the signal measured by the electrode remains stable and is minimally affected by the pulse signal, as shown in Figure 7b. Two sets of signals could be separated in the output of the multimodal biosensor due to different sensing mechanisms.

To demonstrate the performance of the sensor across different populations, we conducted human motion experiments with three volunteers to validate the sensor's broad applicability. Volunteer 1 is an ordinary healthy male, and Volunteer 2 is an ordinary healthy female, and Volunteer 3 is a healthy male who exercises regularly. The test results for Volunteer 1 are shown in Figure 7c,d, where the pulse wave profile and sweat glucose levels gradually change with the continuation of exercise. With the increase of exercise load, the body needs more blood to deliver oxygen, and the amplitude of ventricular contraction increases to enhance blood supply. Therefore, the percussion (P) wave gradually increases.^[^ ^51^ ^]^ The tidal (T) wave first climbs up and then decreases (Figure S17, Supporting Information), which may be attributed to relatively tense vessels and the resistance increment to blood flow propagation at the beginning of exercise. However, as the exercise load continues to increase, vessels gradually expand and become smooth, resulting in a decrease in blood flow propagation resistance. So the tidal wave gradually decreases and disappears, indicating that Moderate Pulse becomes Smooth Pulse after exercise. At the same time, due to the relaxation of muscle vessels during exercise, peripheral resistance decreases, resulting in a decrease in the rising rate of the pulse ascending process.^[^ ^52^ ^]^ The dicrotic (D) wave climbs up slightly at the beginning, and no significant signal variation is observed in subsequent changes, which is in agreement with some published studies.^[^ ^53^ ^]^ This is because an increase in heart rate may lead to an increase in D wave during exercise. Apart from this, the relaxation of skeletal muscle vessels declines the D wave, resulting in an overall stability.^[^ ^54^ ^]^ The improved muscle metabolism can also cause muscle vasodilation and decrease muscle vascular resistance, inducing a decline in the dicrotic notch.^[^ ^55^ ^]^ In the electrical signal shown in Figure 7d, the glucose concentration in sweat descended from 0.212 mM to 0.097 mM, and gradually stabilized after stopping exercise, which was consistent with the measurement by using a commercial blood glucose meter. Fluctuations during movement may be caused by slight changes in resistance during low concentration tests (Figure S18, Supporting Information). Additionally, the pulse rate recovered from a maximum of 139.8 beats per min (b.p.m.) to 75.6 b.p.m. within 30 min with the cessation of movement, as shown in Figure 7e. The pulse variation results measured for Volunteers 2 and 3 are similar to those of Volunteer 1 (Figure S19, Supporting Information). However, in Volunteer 3, the pulse profile shows a smaller tidal peak before exercise, and after exercise, the tidal peak decreases rapidly, while the changes in percussion and frequency are not as pronounced as those in Volunteers 1 and 2. These differences may be attributed to Volunteer 3′s regular exercise, which leads to lower vascular resistance in daily life and a quicker adaptation after exercise. In addition, the changes in glucose in Volunteer 2′s sweat were 0.235 mM down to 0.113 mM, and 0.228 mM down to 0.104 Mm in Volunteer 3, which are similar to the results of Volunteer 1. These results have important clinical significance for daily monitoring of diabetic patients.

## 3 Conclusion

In summary, we have demonstrated an optoelectronic hybrid multimodal wearable optical fiber sensor which can simultaneous monitor biomechanics and glucose. This seamless integrated multimodal sensor is firstly fabricated by laser patterning on the PDMS with porous LIG to form the interdigital electrode, which also modulates the refractive index of the microfiber encapsulated into PDMS, building up a mLPG. To achieve accurate multi‐parameter sensing, two different sets of electrical and optical signals were integrated in one LIG‐mLPG sensor with distinct operation mechanisms, and comprehensive characterizations were performed to validate the pressure sensing viability and the electrical response of graphene electrode of the LIG‐mLPG sensor. As the flexible electrode and functionalized with the conductive GB hydrogel, a glucose biosensor is constructed by loading glucose oxidase into the hydrogel. Compared with existing multimodal sensors, LIG‐mLPG not only enhances the functionality and compactness, effectively saves space, but also maintains fast response time, high sensitivity and stability (Table S1, Supporting Information). Meanwhile, the mLPG triggers mode coupling with resonant wavelength, which can be concurrently served as a spectrally‐resolved optical sensor for biomechanics monitoring. Furthermore, we thoroughly study and verify the independence of two sensing mechanisms upon different stimuli, and further demonstrate the detection of glucose in wound exudate of rat model and simultaneous monitoring of artery pulse and sweat glucose in human model. Regarding further improvements to the current sensor, we can integrate two readout systems for optical and electrical signals into a single demodulation system through external rigid circuit modules for better flexibility and comfort characteristics in future wearable devices.^[^ ^56^ ^]^ Moreover, the electrical properties of the obtained LIG can be optimized by modify the PDMS substrate, such as doping PDMS to enrich its carbon sources, which can improve the conductivity of LIG.^[^ ^57^ ^]^ Developing hydrogels with better moisture retention systems and optimizing hydrogel elution methods can also help enhance the long‐term usability of the wearable sensor.^[^ ^58^ ^]^ For future applications, this sensor offers a new design concept for compact and multifunctional wearable sensors that enable reliable measurements for various hybrid stimuli monitoring and provides better understanding on glucose levels and human biomechanical parameters for optimized management of diabetes.

## 4 Experimental Section

### Experimental Materials

PDMS (Sylgard 184, Dow Corning) was purchased from Guangzhou Zock Biotechnology Development Co., Ltd. Guanosine, boric acid (B(OH)_3_), ascorbic acid (AA), lactic acid, and uric acid (UA) were all purchased from Aladdin Biochemical Technology Co., Ltd. (Shanghai, China). Sodium hydroxide (NaOH) solution is provided by Guangzhou Chemical Reagent (China). HEPES buffer solution was purchased from Macklin Biochemical Technology Co., Ltd. (Shanghai, China). GOx was purchased from Guangzhou Baohui Biotechnology Co., Ltd. Glucose was purchased from West Asia Chemical Co., Ltd. (Shandong, China).

### Fabrication of LIG‐mLPG

Figure S2, Supporting Information shows of the schematic of the setup for the fabrication of the LIG‐mLPG. Firstly, a microfiber was fabricated by drawing a standard single mode fiber (SMF) down to micron scale by using the flame‐brushing technique.^[^ ^31^ ^]^ The SMF was clamped and fixed on two horizontal motors, and the hydrogen oxygen flame nozzle scanned to soften the SMF. Simultaneously, two horizontal motors slowly moved to pull and taper the melted SMF, with the fiber diameter shrinking from 125 to ∼8 µm via precise control. Then, the PDMS was used for the encapsulation of microfibers. The commercial PDMS was prepared by mixing prepolymer A and curing agent B in a ratio of 10:1, which was stirred evenly until a large number of small bubbles appeared in the PDMS, followed by the centrifugal treatment to remove the bubbles. Subsequently, the collected PDMS was dropped onto a glass slide and flattened by a coating machine to form a substrate layer with a thickness of ∼200 µm. The fabricated microfiber was then carefully submerged into the uncured PDMS substrate. Afterward, the sample was placed in a drying oven (80 °C) for 30 min to cure PDMS and secure the microfiber from breakage or bending deformation. The cross‐section of microfibers encapsulated in PDMS was shown in Figure S1, Supporting Information. The encapsulated microfiber sample was ultimately fixed onto a laser engraving system with laser wavelength of 532 nm. The laser‐induced patterning was designed as a series of rectangular array, which was also formed as the modulation structure of the LIG‐mLPG with the period of 500 µm. With a scanning speed of 2 mm s^−1^, the laser‐induced patterning of PDMS was performed, modulating the refractive index of the microfiber and forming a LIG‐mLPG.^[^ ^37^ ^]^

### Preparation of Flexible Optoelectronic Hybrid Multimodal Sensor

The preparation process of GB hydrogel is similar with the method in Ref. [59] The molten GB hydrogel was transferred onto LIG‐mLPG, which subsequently cooled down and solidified to form GB hydrogel film, ultimately constituting the GB hydrogel/LIG‐mLPG structure. To load GOx into GB hydrogel, the HEPES buffer solution containing 1 mg/mL GOx (10 × 10^−3^ M, pH 7.4, sodium chloride in 50 × 10^−3^ M) was used to soak the hydrogel for 1h.^[^ ^60^ ^]^ Glucose reagents with different target concentrations were added into the hydrogel, and a multimeter (KEITHLEY DMM7510) was applied to measure the resistance variation of the GB hydrogel.

### Characterization of the LIG and GB Hydrogels

The physical photo of the LIG‐mLPG was recorded by a CCD camera. The structure and morphology of LIG and GB hydrogels were observed by SEM (ZEISS, ULTRA‐55, Germany). The peak values of PDMS and LIG chemical bonds were measured by FTIR (Vertex 70v, Bruker tensor 27, Germany). The Raman spectra of LIG were recorded by using confocal Raman spectroscopy with an excitation wavelength of 785 nm (DXR, ThermoFisher Scientific, USA). The elemental information of LIG was tested by K‐Alpha XPS (ESCALAB 250XI, ThermoFisher Scientific, USA). The static contact angle was measured by a dynamic contact angle tester (JY‐82B Kruss DSA, Dataphysics, Germany). The rheological characteristics were tested by a rotational rheometer (Kinexus Pro, Malvern, UK) with a parallel plate geometry with diameter of 20 mm.

### Pressure Sensing Performance

Two ends of the prepared LIG‐mLPG were connected with a broadband light source (Golight 1250–1650 nm) and a spectrometer (YOKOGAWA, AQ6370D), respectively, while the positive and negative terminals of the LIG interdigital electrode are connected to the positive and negative terminals of a multimeter (KEITHLEY DMM7510). First, the LIG‐mLPG was clamped at both ends using a fixture, allowing the sensor portion in the middle to maintain parallel. Then, a digital push‐pull force gauge was used to apply pressure to the LIG‐mLPG, recording the corresponding spectral changes of the mLPG as well as the resistance changes of the LIG. The response time was measured using spectral intensity detection technology. Additionally, the I‐V curve was measured by using a KEITHLEY 2614B source meter.

### GB Hydrogel Performance

To investigate the effects of different factors on the mechanical properties of GB hydrogel, it was placed in a rheometer, where various frequency scans and strain values were applied to measure the corresponding (G') and (G″). Additionally, glucose solutions of varying concentrations, artificial sweat and simulated wound fluid were added to the rheometer to measure their corresponding (G′) and (G″), validating the mechanical properties in different environments. After waterproof encapsulation of the non‐sensing area of the LIG with PDMS, the GB hydrogel was modified into the LIG interdigital electrode and immersed in saline solution. Glucose solutions of appropriate concentrations are added to the prepared solution, and a multimeter (KEITHLEY DMM7510) was used to connect the positive and negative terminals of the LIG interdigital electrode for resistance testing.

### Construction of Diabetes Rat Model

6 to 8‐week‐old female CD (SD) IGS rats were purchased from Charles River (Foshan, China). The animal experiment was approved by the Professional Committee of Experimental Animal Ethics of Jinan University (Approval No.: IACUC‐20241101‐19), and all rats were housed in the Experimental Animal Center of Jinan University. Type 1 diabetes rats were induced by a single intraperitoneal injection of streptozotocin (STZ, 55 mg kg^−1^). 1 week after the administration of STZ, the rats were considered to suffer from diabetes when the blood glucose level of the rats exceeded 16.7 mmol L^−1^. During the whole process, the blood glucose levels of rats were recorded through tail vein blood by a commercial blood glucose meter. After successful modeling, 1% pentobarbital sodium was injected intraperitoneally into rats for anesthesia and hair removal treatment. After cleaning with iodine, a 25 mm × 25 mm wound was cut with scissors. Healthy rat wounds are treated in the same way.

### Real Time Monitoring of In Situ Wounds and In‐situ Motion

A small animal anesthesia machine (R580S, RWD Life Science Co. Ltd., China) was used to introduce the isoflurane into the body of the rats under test, with a flow rate of 0.5 L min^−1^ and a density of 1.5%. During the experiment, animal insulation blankets were used to maintain temperature stability in rats. The LIG‐mLPG device modified with GB hydrogel was attached to the wound of rats. Then, the diabetes rats were intraperitoneally injected with insulin, while the healthy rats were not injected with insulin. Similarly, subjects with consent underwent in‐situ testing. The LIG‐mLPG sensor was connected to the wrist or chest of each subject. The subjects exercised on a spinning bike for 120 min, maintaining a constant speed during the exercise. The grating signal was collected by spectral intensity detection technique, and the resistance change of GB hydrogel was measured by a multimeter (KEITHLEY DMM7510). The corresponding hardness and blood glucose reference standards were collected every 30 min by commercial Shore hardness tester and commercial blood glucose meter, respectively.

## Conflict of Interest

The authors declare no conflict of interest.

## Supporting information



Supporting Information

## Data Availability

The data that support the findings of this study are available from the corresponding author upon reasonable request.
