# Supplementary material for: Flexible Optoelectronic Hybrid Microfiber Long‐period Grating Multimodal Sensor
Source: Adv Sci (Weinh). 2025 Mar 8;12(17):2501352. doi: 10.1002/advs.202501352 (PMC12061331; doi:10.1002/advs.202501352)
Supplement: Supplementary file 1 — Supporting Information [file ADVS-12-2501352-s001.docx]

Supporting Information

Flexible optoelectronic hybrid microfiber long-period grating multimodal sensor

Zhenru Li, Li-Peng Sun,* Yanzhen Tan, Zhiwei Wang, Xiao Yang, Tiansheng Huang, Jie Li,* Yi Zhang,* and Bai-Ou Guan*

***** **Table of Contents**

1. Cross-sectional SEM images of the microfiber embedded in the PDMS. (Figure S1)
2. Fabrication process of the LIG-mLPG. (Figure S2)
3. Morphology, width, and depth of LIG under different laser powers and scanning speeds. (Figure S3 and S4)
4. Raman spectra of LIG at different scanning speeds. (Figure S5)
5. The wavelength stability of the LIG-mLPG within 1 hour. (Figure S6)
6. Hydrophobic angle of the PDMS, LIG and the LIG after hydrophilic treatment. (Figure S7)
7. Cyclic voltammetry and Electrochemical impedance spectroscopy tests of graphene interdigital electrode. (Figure S8 and S9)
8. Stability of the graphene electrode. (Figure S10)
9. SEM of GB hydrogel structure. (Figure S11)
10. Mechanical property of GB hydrogel at different glucose concentrations and real body fluid environment obtained by rheological test. (Figure S12)
11. The response of wavelength shifts of mLPG to glucose. (Figure S13)
12. Optical and Electrical sensors cycle stability test. (Figure S14 and S15)
13. Spectral response of LIG-mLPG attached with different hardness objects under the same pressure. (Figure S16)
14. Pulse changes with exercise load. (Figure S17)
15. Comparison of glucose detection results under pressure and non-pressure conditions. (Figure S18)
16. Changes in the pulse wave and sweat glucose concentration on the wrist during exercise of volunteer 2 and 3. (Figure S19)
17. Table 1: Comparison with existing multimodal sensors.


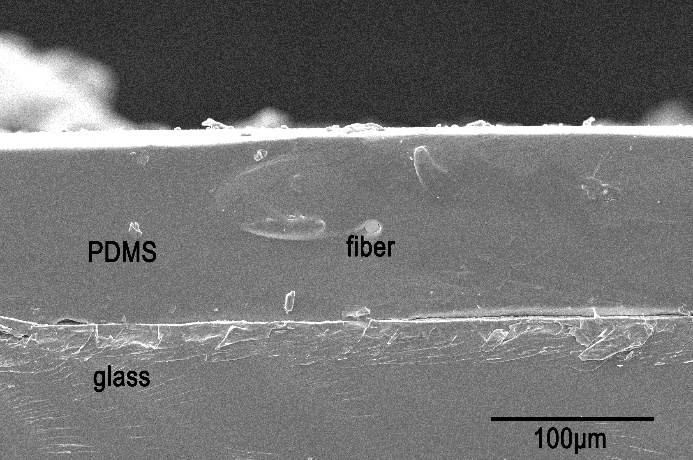


**Figure S1:** Cross-sectional SEM image of the microfiber embedded in the PDMS.


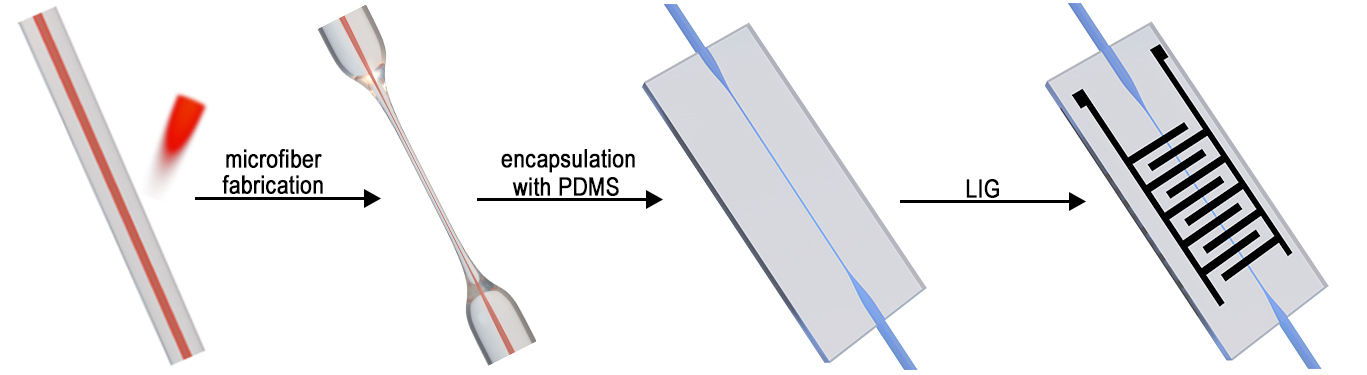


**Figure S2:** Fabrication process of the LIG-mLPG.


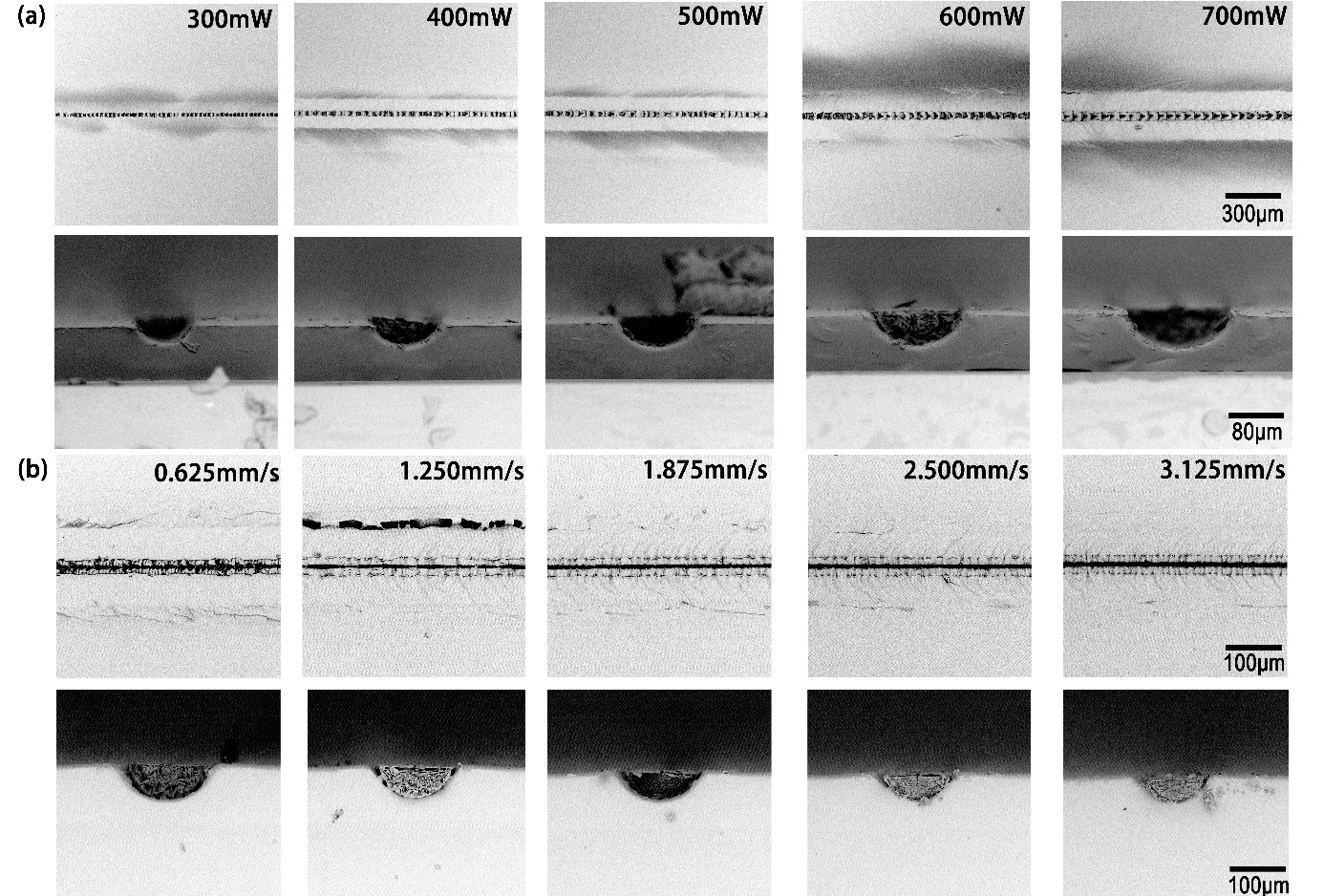


**Figure S3:** Cross-sectional morphology of LIG (a) with different laser powers under scanning speed of 1.250 mm s^-1^; (b) with different scanning speeds under laser power of 600 mW.


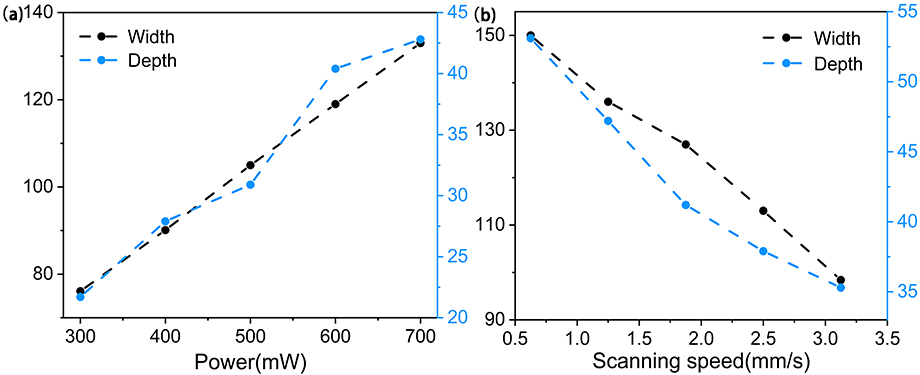


**Figure S4:** The width and depth of LIG (a) with different laser powers under scanning speed of 1.250 mm s^-1^; (b) with different scanning speeds under laser power of 600 mW.
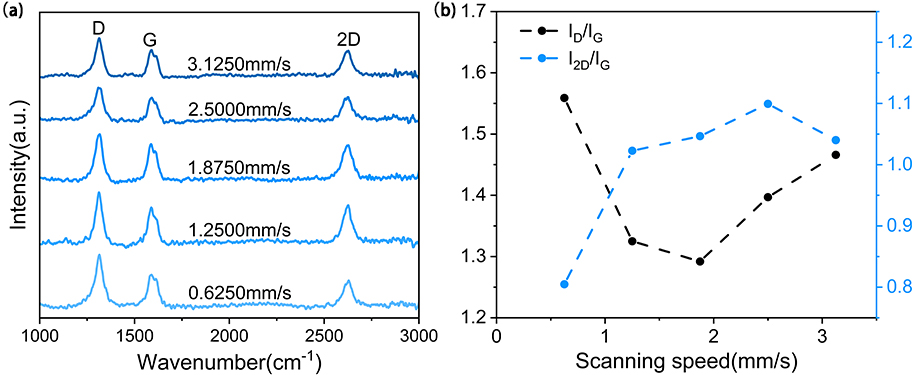


**Figure S5:** (a) Raman spectra of LIG at different scanning speeds; (b) The ratio of the I_D_ peak to the I_G_ peak (black) and the ratio of the I_2D_ peak to the I_G_ peak (blue) changes with scanning speed.


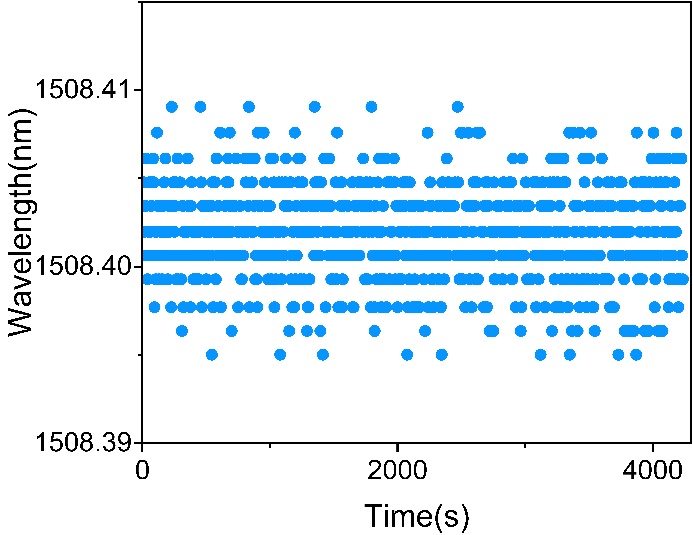


**Figure S6:** The wavelength stability of the LIG-mLPG.


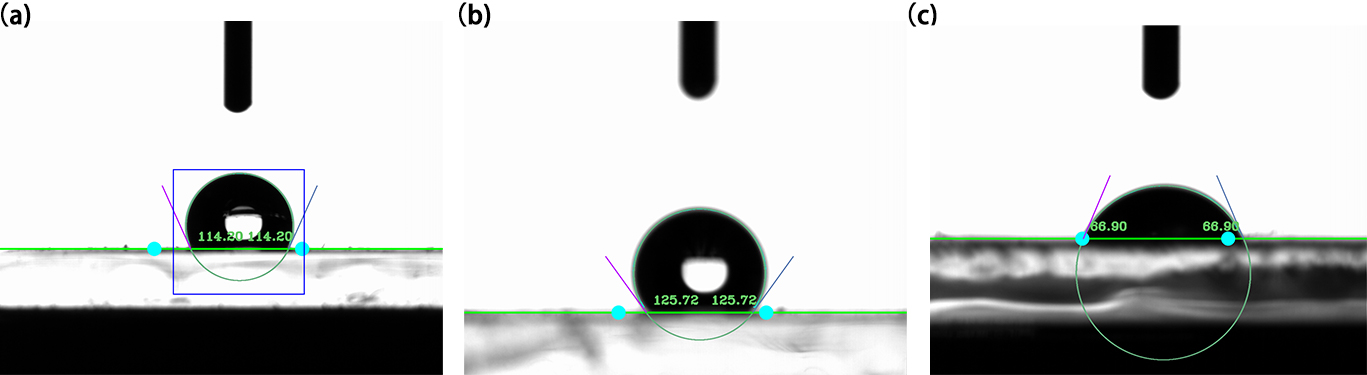


**Figure S7:** (a) Hydrophobic angle of the PDMS; (b) Hydrophobic angle of the LIG before hydrophilic treatment; (c) Hydrophobic angle of the LIG after hydrophilic treatment.

Figure S7a shows that the hydrophobic angle of PDMS is 114.20°. After laser patterning, the hydrophobic angle of LIG increases to 125.72°, as shown in Figure S7b. Therefore, it is difficult for the liquid to enter the deep of porous LIG and contact the evanescent field leaked by mLPG, so the refractive index response to the liquid environment is very low. After the hydrophilic reagent (Mesophilic-2000, purchased from MesoBioSystem, Wuhan, China) and isopropanol being mixed in a ratio of 7:3, the LIG was immersed in the mixture for 2 min for hydrophilic treatment, and the hydrophobic angle was measured to be 66.90° after drying with an air gun^[1][2]^, as shown in Figure S7c. The liquid is more likely to contact the evanescent field of mLPG, and the refractive index response of mLPG to the liquid environment increases. In order to reduce the refractive index response in the liquid environment, we chose LIG that was not treated with a hydrophilic reagent for subsequent experiments.


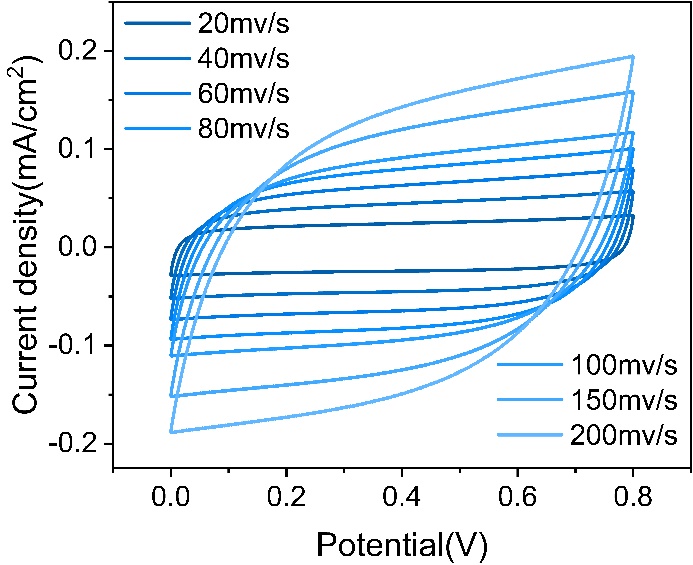


**Figure S8:** Cyclic voltammetry test of graphene interdigital electrode at different scan rates.


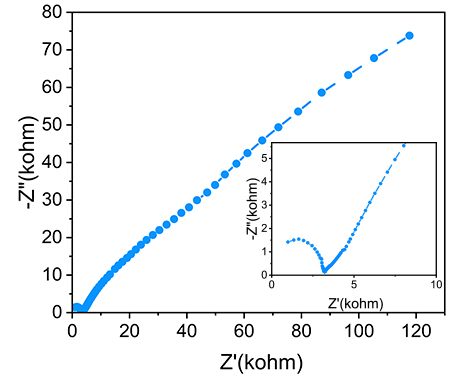


**Figure S9:** Electrochemical impedance spectroscopy test of the graphene interdigital electrode.


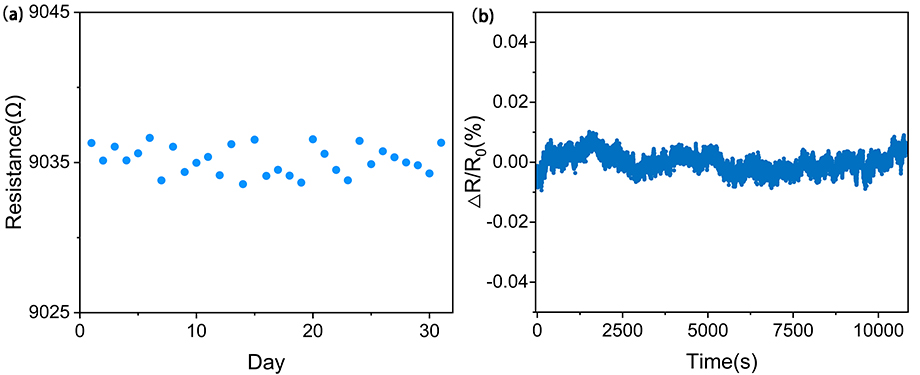


**Figure S10:** Stability of the graphene electrode (a) for 1 month and (b) for 3 h.


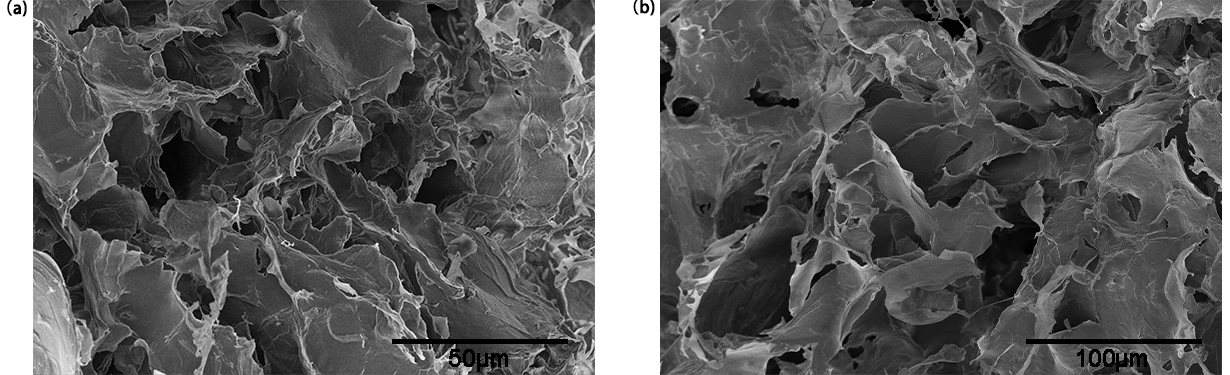


**Figure S11:** SEM images of the GB hydrogel.


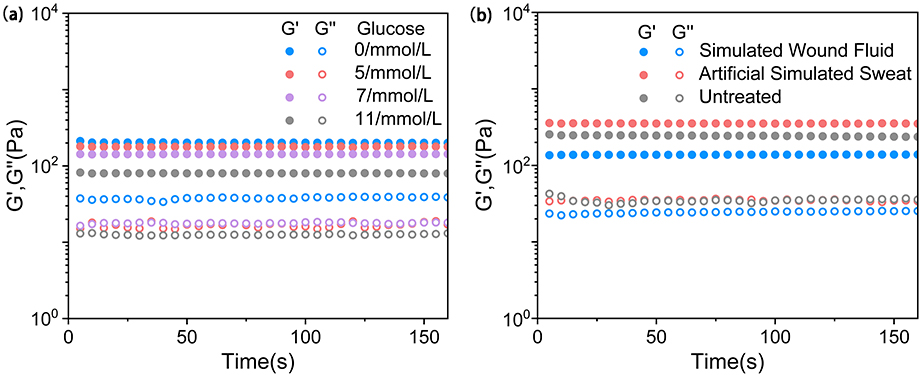


**Figure S12:** Mechanical property of GB hydrogel at (a) different glucose concentrations; (b) real body fluid environments obtained by rheological test.


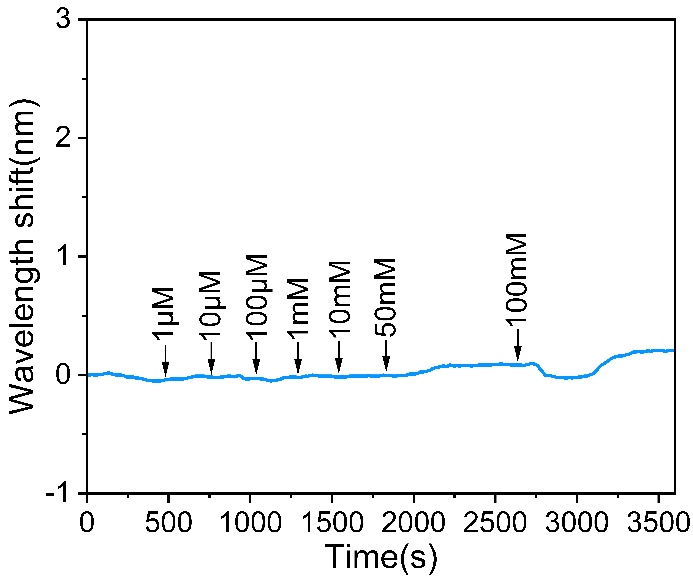


**Figure S13:** The response of wavelength shifts of mLPG to glucose.


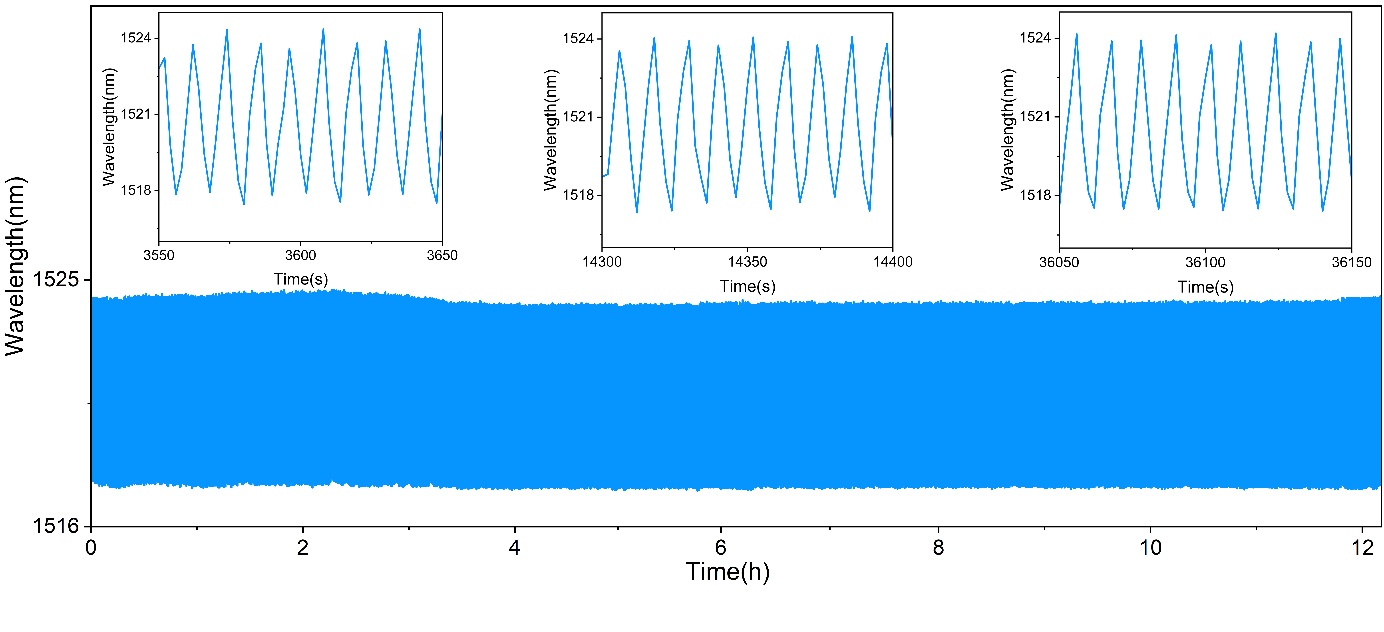


**Figure S14:** Optical sensor cycle stability test.

**
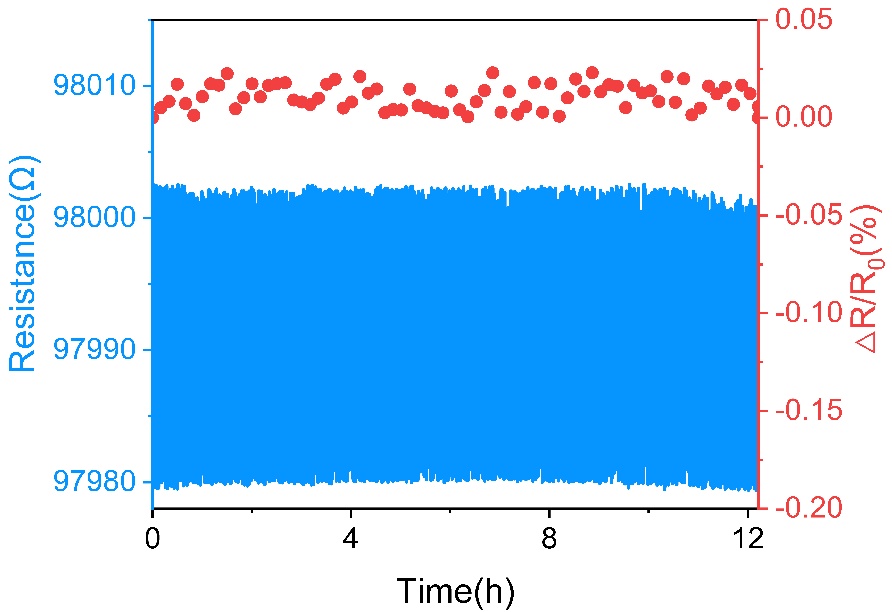
**

**Figure S15:** Electrical sensor cycle stability test.


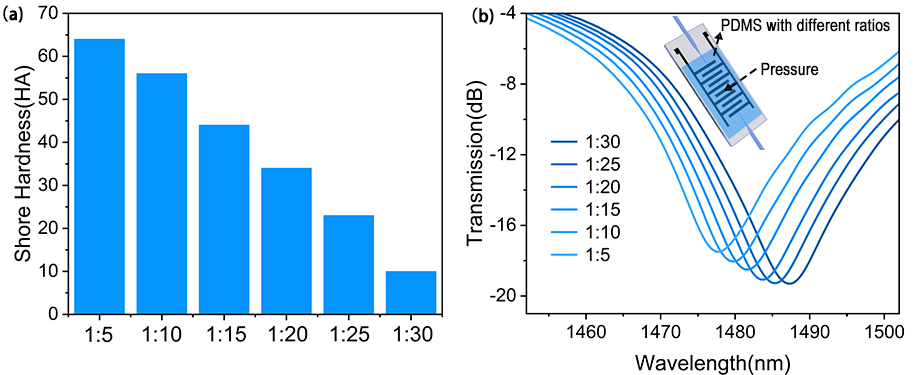


**Figure S16:** (a) The hardness of PDMS with different ratios; (b) Spectral response of LIG-mLPG attached with different ratios of PDMS under the same pressure (5kPa).

In order to verify the pressure response of LIG-mLPG attached with objects of different hardness, we prepared PDMS with different hardness, as shown in Figure S16a. When PDMS was attached to the surface of LIG-mLPG, the same pressure (5 kPa) was used for testing, and different spectral responses were obtained, as shown in Figure S16b, indicating that LIG-mLPG can recognize objects of different hardness.


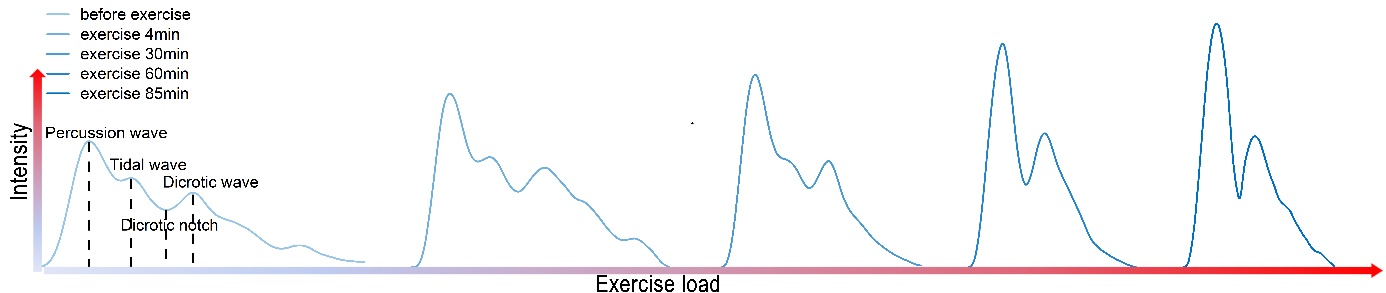


**Figure S17:** Pulse changes with exercise load.


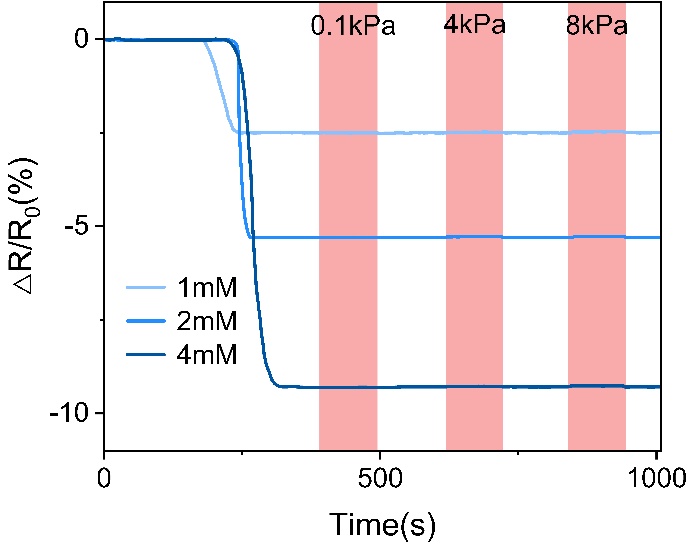


**Figure S18:** Changes in glucose detection at 0.1, 4 and 8 kPa

Under pressure conditions, the deformation causes a slight increase in resistance, which results in a lower glucose measurement accuracy that is inversely proportional to the change in resistance. Therefore, as the pressure increases, the glucose measurement value decreases. Within the linear detection range, a pressure of 0.1-8 kPa corresponds to a decrease in glucose concentration of approximately 0.002 to 0.027 mM, which has a more significant impact on applications involving low glucose concentrations. This effect may potentially be offset by subsequent concentration compensation for the measured decrease at specific pressure levels.


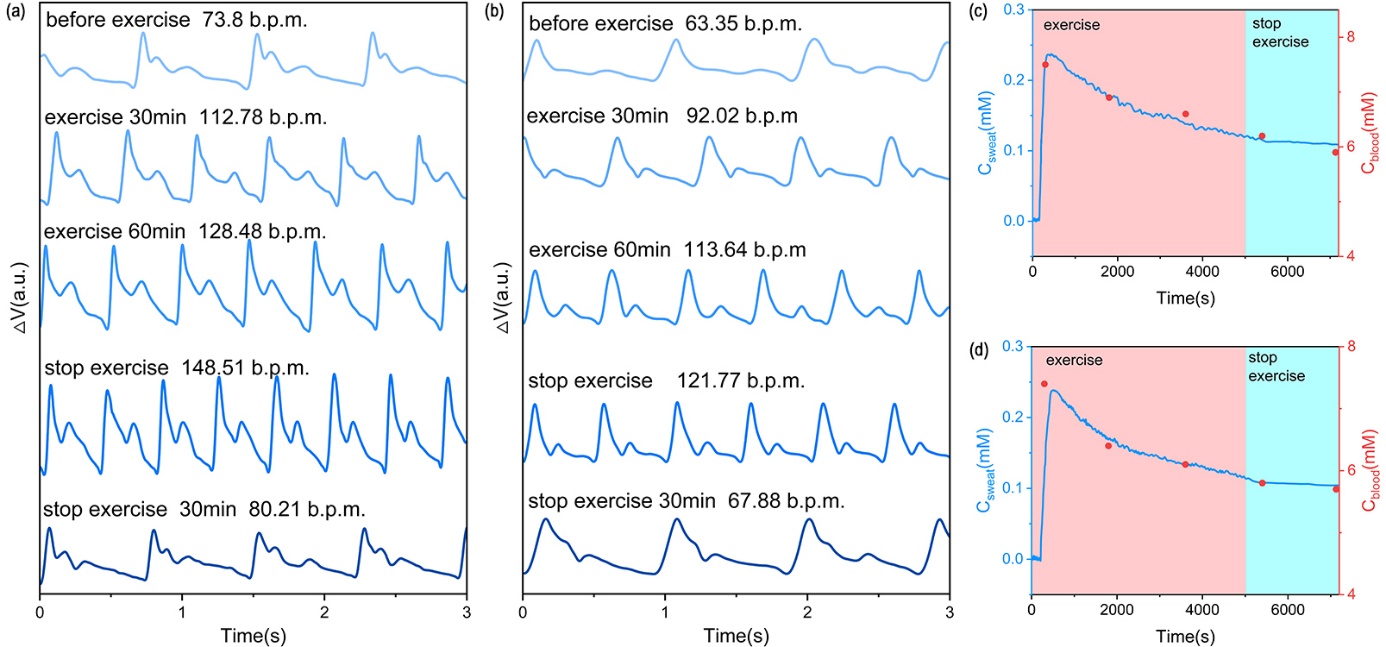
**Figure S19:** Changes in the pulse wave on the wrist during exercise of (a) Volunteer 2 and (b) Volunteer 3; Changes in glucose concentration in sweat (blue) and blood glucose measurement with a commercial blood glucose meter (red) during exercise of (c) Volunteer 2 and (d) Volunteer 3.

**Table 1. Comparison with existing multimodal sensors**

| Preparation  technology | Sensing function | Sensitivity | Response time | Stability | Ref. |
| --- | --- | --- | --- | --- | --- |
| Fully inkjet-printed | Temperature | NA | 0.45 s | 100 cyclic | [3] |
|  | Pressure | 0.03%/ kPa | 40 ms | 10000 cyclic |  |
| Ultrasonication anchoring | Temperature | -0.745 %/℃ | 150 s | NA | [4] |
|  | Pressure | 0.152 kPa/nF | 110 ms | 7000 cyclic |  |
| 3D printing | Temperature | 1.447 nm/℃ | NA | NA | [5] |
|  | Force | 56.35 nm/μN |  |  |  |
| [Solution casting](https://www.sciencedirect.com/topics/engineering/casting-solution) and in situ reduction | Strain | 1.6 /∆ε | 90 ms | 200 cyclic | [6] |
|  | Temperature | −0.076 /℃ | NA | 5 cyclic |  |
|  | Humidity | 0.0225 ∆R/RH% | 25 s | 5 cyclic |  |
| Laser-patterning | Pressure | 0.42 or 0.26 /N | 1 ms | 10000 cyclic | [7] |
|  | Temperature | 7 %/℃ | NA | 600 cyclic |  |
|  | Proximity | NA | 0.05 s | 1600 cyclic |  |
| Molding and thermally curing | Strain | NA | 23 ms | 1000 cyclic | [8] |
|  | Temperature | 0.8 %/℃ | 3 s | 5 cyclic |  |
|  | pH | 27 %/pH | 34 s | 5 cyclic |  |
| Lithography and wet etching | Glucose | 44.56 μA/mM | NA | 20 cyclic | [9] |
|  | pH | 60.1 mV/pH |  | 20 cyclic |  |
|  | ECG | NA |  | NA |  |
| Laser induced graphene | Tyrosine | 4.1 mA /M | NA | NA | [10] |
|  | Uric acid | 27.8 mA/M |  |  |  |
| Bilayer hydrogel | Solid lactate | 93.8 nA/nmol | 400 s< | NA | [11] |
|  | Solid cholesterol | 201.34 nA/nmol |  |  |  |
| Mechanized assembly of sensor arrays | Temperature | 0.21%/℃ | NA | NA | [12] |
|  | pH | 59.7 mV/pH |  |  |  |
|  | Ammonium | 59.7 mV/decade |  |  |  |
|  | Glucose | 16.34 nA/mM |  |  |  |
|  | lactate | 41.44 nA/mM |  |  |  |
|  | Uric acid | 189.6 nA/mM |  |  |  |
| Laser induced graphene | Pressure | 2.06 nm/kPa | 2.8 ms | 4000 cyclic | This  work |
|  | Glucose | 2.57 %/mM | Change with concentration | 4000 cyclic |  |

**Reference:**

[1] F. Zhang, M. Liu, R. Liu, J. Li, Y. Sang, Y. Tang, X. Wang, S. Wang, *Biosens. Bioelectron.* **2021**, 171, 112701.

[2] Y. Huang, R. Zhao, W. Miao, D. Liang, Z. Liu, X. Wei, Z. Liang, *Opt. Lett.* **2022**, 47, 3824.

[3]. T. Yuan, R. Yin, C. Li, C. Wang, Z. Fan, L., Pan, *Chem. Eng. J.* **2023**, 473, 145475.

[4]. K. Keum, J.Y. Kwak, J. Rim, D.H. Byeon, I. Kim, J. Moon, S.K. Park, Y-H, Kim. *Nano Energy*, **2024**, 122, 109342.

[5]. C. Xiong, C. Wang, Y. Qin, R. Yu, W. Ji, A-Q. Liu, Y. Shen, L. Xiao, *ACS Appl. Mater. Interfaces*, **2024**, 16, 30443.

[6]. L. Chen, X. Chang, H. Wang, J. Chen, Y. Zhu, *Nano Energy*, **2022**, 96, 107077.

[7]. S. Wang, X. Wang, Q. Wang, S. Ma, J. Xiao, H. Liu, J. Pan, Z. Zhang, L. Zhang, *Adv. Mater.* **2023**, 35, 2304701

[8]. J. Guo, J. Tuo, J. Sun, Z. Li, X. Guo, Y. Chen, R. Cai, J. Zhong, L. Xu, *Adv. Mater.* **2025**, 37, 2412322.

[9]. M.A. Zahed, D.K. Kim, S.H. Jeong, M.S. Reza, G.B. Pradhan, H. Song, M. Asaduzzaman, J.Y. Park. *ACS sensors*, **2023**, 8, 2960.

[10]. V. Kammarchedu, D. Butler, A. Ebrahimi. *Anal. Chim. Acta*, **2022**, 1232, 340447.

[11]. R.T. Arwani, S.C.L. Tan,A. Sundarapandi, W.P. Goh, Y. Liu, F.Y. Leong, W. Yang, X.T. Zheng, Y. Yu, C. Jiang, Y.C. Ang, L. Kong, S.L. Teo, P. Chen, X. Su, H. Li, Z. Liu, X. Chen, L. Yang, Y. Liu. *Nat. Mater.* **2024**, 23, 1115.

[12]. E. Shirzaei Sani, C. Xu, C. Wang, Y. Song, J. Min, J. Tu, S.A. Solomon, J. Li, J. L. Banks, D.G. Armstrong, W. Gao., *Sci. Adv.* **2023**, 9, eadf7388.
